# Different lysine-to-methionine ratios in a low-protein diet affect the microbiome and metabolome, influencing the jejunal barrier function in Tibetan sheep

**DOI:** 10.3389/fmicb.2025.1441143

**Published:** 2025-02-12

**Authors:** Fengshuo Zhang, Zhenling Wu, Yu Zhang, Quyangangmao Su, Kaina Zhu, Xuan Chen, Shengzhen Hou, Linsheng Gui

**Affiliations:** College of Agriculture and Animal Husbandry, Qinghai University, Xining, China

**Keywords:** Lys/Met ratio, microbiome, metabolome, jejunum, Tibetan sheep

## Abstract

**Introduction:**

The objective of this study was to evaluate the effects of the dietary lysine (Lys)/ methionine (Met) ratio in a low-protein diet on short-chain fatty acid (SCFA) profiles, villus morphology, antioxidant capacity, and immune status of the jejunum in Tibetan sheep.

**Methods:**

A total of 90 weaned Tibetan sheep, each 2 months old with an initial weight of 15.37 ± 0.92 kg, were randomly divided into three treatment groups. These groups were supplemented with different Lys/Met ratios of 3 [low protein-high methionine (LP-H)], 2 [low protein-medium methionine (LP-M)], and 1 [low protein-low methionine (LP-L)] in the basal diet (10% crude protein). The feeding trial lasted 100 days, including a 10-day acclimation period and a 90-day experimental period.

**Results:**

The hematoxylin–eosin (H&E) sections showed that the LP-L group had a significantly increased villus height compared to the LP-M and LP-H groups (*p* < 0.05). In addition, the LP-L group showed higher levels of Superoxide dismutase (SOD) activity and Total Antioxidant Capacity (T-AOC) concentrations (*p* < 0.05). A lower concentration of Interleukin-1 beta (IL-1β) was observed in the LP-H group (*p* < 0.05). The activities of α-amylase, chymotrypsin, and lipase were higher in the LP-L group compared to the LP-H group (*p* < 0.05). Bacterial sequencing showed that both Chao1 and ACE richness were significantly increased in the LP-L group (*p* < 0.05), suggesting that the species richness in the jejunum is connected to the ratio of dietary Lys/Met. Furthermore, lowering the dietary Lys/ Met ratio significantly increased the abundance of *Romboutsia*, the *Ruminococcus gauvreauii* group, the *Lachnospiraceae NK3A20* group, *Ruminococcus 2*, and the *Christensenellaceae R-7* group (*p* < 0.05) while decreasing the abundance of *Methanobrevibacter* (*p* < 0.05). Several differential metabolites, including beta-alanine, pantothenate, pantothenic acid, phosphoenolpyruvate, cysteine, adenosine 5′-diphosphate, isodeoxycholic acid, glutamate conjugated cholic acid, and 3-dehydrocholic acid, were significantly increased in the LP-L group (*p* < 0.05). The functional analysis based on the Kyoto Encyclopedia of Genes and Genomes (KEGG) annotations indicated that these metabolites were mainly involved in pantothenate and CoA biosynthesis, ferroptosis, and the tricarboxylic acid cycle. Several genes related to barrier function, such as Occludin and Muc- 2, were upregulated in the LP-L group (*p* < 0.05), while IL-6 and TNF-α were downregulated (*p* < 0.05).

**Discussion:**

Collectively, our results suggest that the dietary Met/ Lys ratio could affect the jejunal SCFA concentration by modulating the microbial community and regulating metabolism, thereby contributing to jejunal barrier function. Our findings provide a theoretical basis for the application of Lys/Met diet supplementation in the nutritional management of Tibetan sheep, particularly when reducing the dietary crude protein (CP) level.

## 1 Introduction

Tibetan sheep (*Ovis aries*), an ancient domesticated species, is primarily distributed across the Qinghai-Tibet Plateau at altitudes ranging from 3,000 to 5,000 meters (Wu et al., 2024). Over a long period of natural selection, Tibetan sheep have developed a strong ability to adapt to high-altitude environments characterized by low temperature, hypoxia, nutritional deficiencies, and strong ultraviolet radiation. Currently, approximately 14 million Tibetan sheep thrive in Qinghai province, China, providing meat, wool, skin, and other means of livelihood. The high altitude means that Tibetan sheep require more nutrition to survive. Therefore, the normal dietary crude protein (CP) concentration is approximately 14–16% during the growth phase of Tibetan sheep (Zhu et al., 2024). Protein feed ingredients are characterized by relatively high costs. Currently, the livestock industry relies mainly on imports to meet the protein feed requirement in China. This situation poses a challenge in managing feed prices, which have shown a tendency to rise steadily (Tao et al., 2021). In addition to these economic considerations, plant protein sources (i.e., soybean) are vital for the nutritional needs of both humans and livestock (Cheng et al., 2024), thereby creating competition between these groups for access to these resources. Moreover, reducing the protein level in ruminant diets could substantially improve environmental sustainability by lowering nitrogen excretion (Cortese et al., 2019).

Lysine (Lys)participates in protein biosynthesis, thereby modulating various physiological activities, such as nutrition metabolism, hormone production, and immune function (Yin et al., 2018). Adding Lys at low-protein (LP) levels to the diet was shown to improve the average daily gain by reducing nitrogen excretion and enhancing nitrogen efficiency in Holstein bulls (Zou et al., 2023). Supplementation with Lys was demonstrated to improve microbial-N and the efficiency of microbial protein synthesis in the rumen and alter serum glucose and glucagon concentrations in Tan lambs (Liu et al., 2021b). Similarly, dietary distiller’s dried grains with soluble (DDGS) supplemented with Lys reportedly influences dry matter intake, apparent dry matter digestibility, and nitrogen utilization, resulting in increased serum glucose and albumin in Hu sheep (Chen et al., 2021). Similar to Lys, methionine (Met) serves as a modulator for DNA methylation, protein synthesis, inflammatory response, and antioxidant balance (Velazquez et al., 2020). Lambs that were given an LP diet supplemented with Met exhibited significantly decreased hepatic fat deposition by inhibiting the expression of obesity-associated protein and alkB homolog 5 (Gebeyew et al., 2022). Supplementation of Met with fattening steers during high-temperature seasons reduced feed conversion, increased average daily gain, and enhanced the redness of the *longissimus* muscle (Park et al., 2020). By enhancing intestinal absorption, Met products in diets increased milk performance in dairy cows (Richards et al., 2023). Therefore, we hypothesized that intestinal health might be related to dietary EAAs in Tibetan sheep.

Lys and Met are the top two limiting amino acids (LAAs) for ruminants (Zhao et al., 2019). Accumulating evidence has revealed that supplementation with Lys and Met alone or in combination affects economic performance and organismal health in livestock breeding (Peng et al., 2020). The hepatic nitrogen utilization rate and the serum insulin-like growth factor (IGF-1) content were reported to be significantly higher with an increasing dietary Met/Lys ratio, thereby improving the average daily gain in Holstein bulls (Zou et al., 2023). In our previous study, an appropriate increase in the Met/Lys ratio in the diet had a positive effect on hepatic antioxidant capacity, immune status, and glycolytic activity by regulating the expression of related genes in Tibetan sheep (Ji et al., 2024). Additionally, the Met/Lys ratio of 1:1 in low-protein diets showed superior antioxidant status and cellulase activity in the rumen by modulating the microbiota (*Rikenellaceae RC9 gut group flora* and *Succiniclasticum*) and metabolism (phosphoric acid, pyrocatechol, hydrocinnamic acid, benzamide, and L-gulono-1,4-lactone) of Tibetan sheep (Zhang F. et al., 2024). Therefore, dietary Lys and Met supplementation has exhibited a synergistic effect. However, there is limited comparative research on the effects of Lys and (or) Met supplementation on the intestinal morphology and microflora in Tibetan sheep fed an LP diet.

The jejunum plays a pivotal role in nutrient utilization, immune responses, and electrolyte balance by absorbing essential nutrients (such as fructose, vitamins, glucose, small peptides, and amino acids), which contribute to organismal health and growth performance in ruminants (Gaowa et al., 2021). We hypothesize that the supplementation of lysine (Lys) and methionine (Met) in LP diets will enhance the intestinal health of Tibetan sheep, leading to improved nutrient absorption and overall performance. In this study, we explored the effects of different ratios of Lys/Met in an LP diet on jejunal barrier function in Tibetan sheep. In addition, the potential functional links between jejunal microbiota, metabolites, and phenotypes (e.g., short-chain fatty acid (SCFA) profiles, antioxidant capacity, immune levels, and digestive enzyme activity) were assessed.

## 2 Materials and methods

### 2.1 Experimental design

The feeding trial was conducted from 1 May 2022 to 8 August 2022 in Haiyan County, Qinghai Province, China (Coordinate 36°44 N′, 100°23E′. Altitude: 3,000 m. Humidity: 54%). A total of 90 two-month-old weaned Tibetan sheep, each with a initial weight of 15.37 ± 0.92 kg, were obtained from a commercial Tibetan sheep farm. The Tibetan sheep were randomly divided into three treatment groups, which were supplemented with Lys/Met ratios of 3 [low protein-high methionine (LP-H)], 2 [low protein-medium methionine (LP-M)], and 1 [low protein-low methionine (LP-L)] in the basal diet. The feeding trial continued for 100 days, including a 10-day acclimation period and a 90-day experimental period. The daily feed was divided into two equal meals, which were provided to each sheep at 7:00 and 17:00. All the sheep had ad libitum access to fresh water. The three feedstuffs were prepared as a total mix, consisting of 30% forage and 70% concentrate on a dry matter basis. The basal diet composition and nutrient levels are listed in Table 1.

**Table 1:** Dietary concentrate composition and nutrient levels (dry matter basis).

Items	LP-L	LP-M	LP-H
Ingredient (%)			
Oat hay	15.00	15.00	15.00
Oat silage	15.00	15.00	15.00
Corn	36.53	37.10	37.10
Wheat	7.70	7.70	7.70
Soybean meal	0.70	0.70	0.70
Rapeseed meal	7.00	7.00	7.00
Cottonseed meal	0.70	0.70	0.70
Maize germ meal	0.70	0.70	0.70
Palm meal	11.20	11.20	11.20
NaCl	0.35	0.35	0.60
Limestone	0.35	0.44	0.70
Baking soda	0.07	0.00	0.07
Premix^a^	2.94	2.94	2.94
Lys	1.39	0.93	0.48
Met	0.37	0.24	0.11
Total	100.00	100.00	100.00
Nutrient levels			
DE (MJ/kg)^b^	10.76	10.84	10.84
Crude protein	9.94	9.98	9.98
Ether extract	2.85	2.87	2.87
Crude fiber	22.47	22.61	22.61
Neutral detergent fiber	33.72	33.77	33.77
Acid detergent fiber	23.37	23.39	23.39
Ca	0.42	0.42	0.42
P	0.17	0.17	0.17

a Provided per kilogram of the diets: Cu 15 mg, Fe 55 mg, Zn 25 mg, Mn40 mg, Se 0.30 mg, I 0.5 mg, Co 0.20 mg, VA 20,000 IU, VD 4,000 IU, and VE 40 IU.

b Digestive energy is the calculated value, while the rest are measured values.

### 2.2 Sample collection

At the end of the experiment, 18 Tibetan sheep (*n* = 6 per treatment) were selected randomly for slaughter at a commercial slaughterhouse. Both the tissue and contents of the jejunum were collected. Approximately 2 cm of the middle section was cut and fixed in a 4% paraformaldehyde solution for morphological analysis. In addition, the contents of the jejunum were collected, snap-frozen in liquid nitrogen, and stored at −80°C for future analysis.

### 2.3 Histological analysis

The jejunal tissue was dehydrated, trimmed, dipped in wax, embedded, sectioned, and then stained with hematoxylin–eosin (H&E). The randomly selected morphologically intact target areas of the jejunum were observed using a digital microscope (DP2-BSW, Olympus Corporation, Tokyo, Japan). The villus height and width, mucosal thickness, and corresponding crypt depth were determined at 400 × and 100 × magnification.

### 2.4 Enzyme-linked immunosorbent assay

The jejunal contents were stored in liquid nitrogen for the detection of antioxidant capacity (T-AOC, SOD, GSH-P, CAT, and MDA), immune levels (IgA, IgG, IgM, TNF-α, and IL-1β), and digestive enzyme activities (α-amylase, chymotrypsin, cellulose, trypsin, and lipase). Commercial ELISA kits (Nanjing Jiancheng Bioengineering Institute, Nanjing, China) were used to determine these indicators following the manufacturer’s instructions.

### 2.5 SCFA quantification

Approximately 0.1 g of the jejunal contents was homogenized in 1.0 mL of distilled water. The mixture was centrifuged at 12,000 g for 10 min at 4°C. Then, 500 μL of supernatant aliquot was mixed with 2 mL of metaphosphoric acid and placed at −20°C for 12 h. The concentration of SCFAs was measured using gas chromatography (GC-2014, Kyoto, Japan) according to a previous method (Gao et al., 2024).

### 2.6 16S rDNA gene sequencing

Microbial DNA in the jejunal contents was extracted using the HiPure Stool DNA Kit (Guangzhou Magen Biotechnology Co., Ltd., Guangzhou, China). The 16S rRNA genes of the V3-V4 region of the bacteria were amplified by PCR using specific primers with a barcode. The primer sequences were 341F (5′-CCTACGGGNGGCWGCAG-3′) and 805R (5′-GACTACHVGGGTATCTAATCC-3′). Amplification products were confirmed with 2% agarose gel electrophoresis and purified using the AxyPrep DNA Gel Extraction Kit (Axygen Biosciences, Union City, United States).

The purified products were pooled and paired-end sequenced on an Illumina NovaSeq600 platform, generating 250 bp paired-end reads. To obtain high-quality clean tags, raw reads were filtered using FASTP (Version 0.18.0) to remove low-quality reads. Subsequently, clean reads were clustered into operational taxonomic units (OTUs) at 97% sequence identity using UCLUST (Edgar et al., 2011). Taxonomic classification of the OTUs was performed using the Ribosomal Data Project classifier, which utilized sets of representative sequences to search the Greengenes version 13_8 database (Wang et al., 2007). To evaluate community diversity, alpha diversity indices, including Chao1, ACE, Shannon, and Simpson, were calculated using QIIME (Version 1.9.1). Beta diversity was estimated using the weighted UniFrac distance and visualized through principal coordinate analysis (PCoA). To further explore the differences in the community structure, permutational multivariate analysis of variance (PERMANOVA) was performed to test for significant group differences, as it is appropriate for non-parametric data distributions.

### 2.7 Liquid chromatography–mass spectrometry metabolomics analysis

Approximately 25 mg of the jejunal digesta from each experimental sheep (per sample) were mixed with 500 μL of an extract solution (methanol: acetonitrile: water = 2:2:1). After mixing with a vortex, the mixture was placed at −20°C for 10 min to precipitate proteins. It was then centrifuged at 13,000 g for 15 min at 4°C. The supernatant was subsequently dried in a vacuum centrifuge. For LC–MS analysis, the samples were re-dissolved in 100 μL of a solvent mixture of acetonitrile and water (1:1, v/v).

Untargeted metabolomics was performed using an UHPLC System (1290 Infinity LC, Agilent Technologies, California, USA) coupled with a quadrupole time-of-flight mass spectrometer (AB Sciex TripleTOF 6600, Shanghai Applied Protein Technology Co., Ltd, shanghai Chin) Chromatography was carried out with an ACQUITY UPLC BEH column (2.1 × 100 mm, 1.7 μm, Waters, Ireland, USA). Raw MS data were converted into mzXML format using ProteoWizard MSConvert.

Principle component analysis (PCA) and orthogonal partial least squares discriminant analysis (OPLS-DA) were performed using the “ropls” package in R (Version 3.3.2). The differential metabolites were defined by Variable Importance in Projection (VIP) values > 1.0 and a *p*-value < 0.05. In addition, the Kyoto Encyclopedia of Genes and Genomes (KEGG) annotations were used to determine which differential metabolites were significantly enriched in metabolic pathways.

### 2.8 Quantitative PCR analysis

Total RNA from the jejunal tissue was extracted using a Total RNA kit (TaKaRa, Dalian, China), following the manufacturer’s instructions. Briefly, 50–100 mg of the tissue was homogenized, and the RNA was isolated via chloroform-isopropanol phase separation, precipitated with ethanol, and resuspended in RNase-free water. RNA quality and concentration were measured using a NanoDrop spectrophotometer. Next, the RNA was reverse transcribed into cDNA using the PrimeScript™ RT Reagent Kit (TaKaRa, Dalian, China) with 1 μg RNA, 4 μL 5× PrimeScript Buffer, 1 μL PrimeScript RT Enzyme Mix, and 1 μL Oligo dT. The reaction was carried out at 37°C for 15 min and inactivated at 85°C for 5 s. qPCR was performed using the Applied Biosystems 7500 Fast Real-Time PCR System (Applied Biosystems, United States) with 10 μL SYBR Premix, 1 μL cDNA, 0.5 μL of each primer (10 μM), and 8 μL water. The amplification conditions were as follows: 95°C for 30 s, followed by 40 cycles of 95°C for 5 s and 60°C for 30 s. The relative gene expression was calculated using the 2^−ΔΔCt^ method with GAPDH as the endogenous control. The annealing temperature and PCR product size are shown in Supplementary Table S1.

### 2.9 Statistical analysis

Using the simple random sampling (SRS) method in the statistical analysis system (SAS) software (Version 9.4), the Tibetan sheep were randomly separated into three treatment groups based on body weight and age. The differences in the villus morphology, SCFA profiles, antioxidant capacity, immune levels, and digestive enzyme activity were analyzed using one-way ANOVA. Furthermore, a non-parametric test coupled with the Kruskal–Wallis test was employed to evaluate the differences in the alpha diversity indices and the relative abundance of core bacteria among the three groups, within a 95% confidence interval. A *p*-value of <0.05 was considered statistically significant. Spearman’s correlation was calculated for the differential jejunal bacteria, differential metabolites, antioxidant capacity, immune levels, and digestive enzyme activities, with correlation heat maps visualized using the R package (Version 3.3.2).

## 3 Results

### 3.1 Effect of the dietary Lys/Met ratios on the jejunal phenotypes

**Figure 1:**
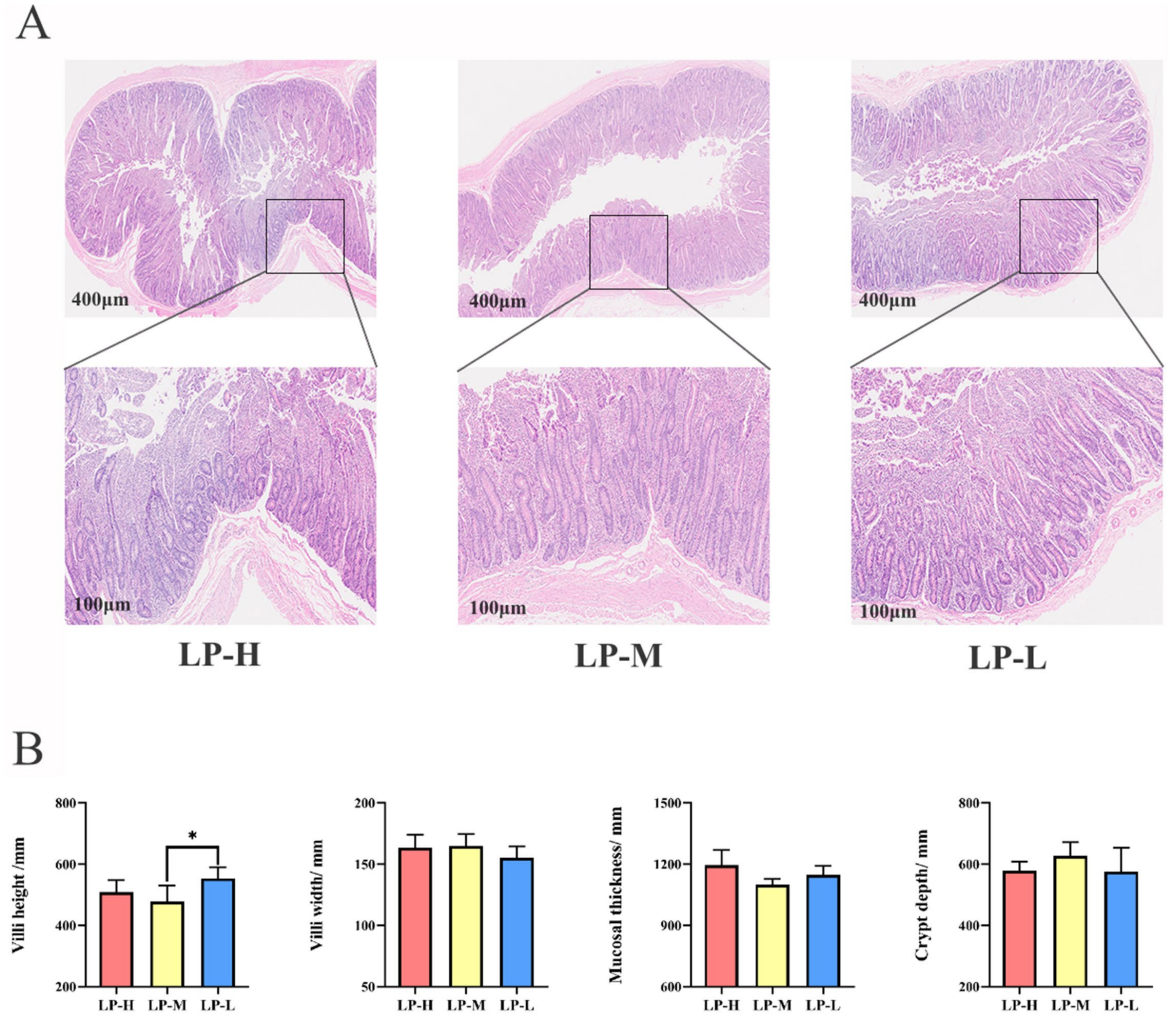
Effect of Lys/Met ratio on jejunal phenotypes. **(A)** Representative histological images of jejunal slide stained with hematoxylin-eosin (original magnification 400× and 100× μm). **(B)** The villi height, villi width, mucosal thickness and corresponding crypt depth of jejunum. Data are presented as mean ± SD. *p < 0.05.

The H&E sections revealed morphological alternations in the jejunal tissue of the Tibetan sheep, including villus height, villus width, mucosal thickness, and corresponding crypt depth (Figure 1A). Compared to the LP-M and LP-H groups, the LP-L group exhibited a significantly increased villus height (*p* = 0.032) (Figure 1B).

### 3.2 Effect of the dietary Lys/Met ratios on the jejunal health

**Figure 2:**
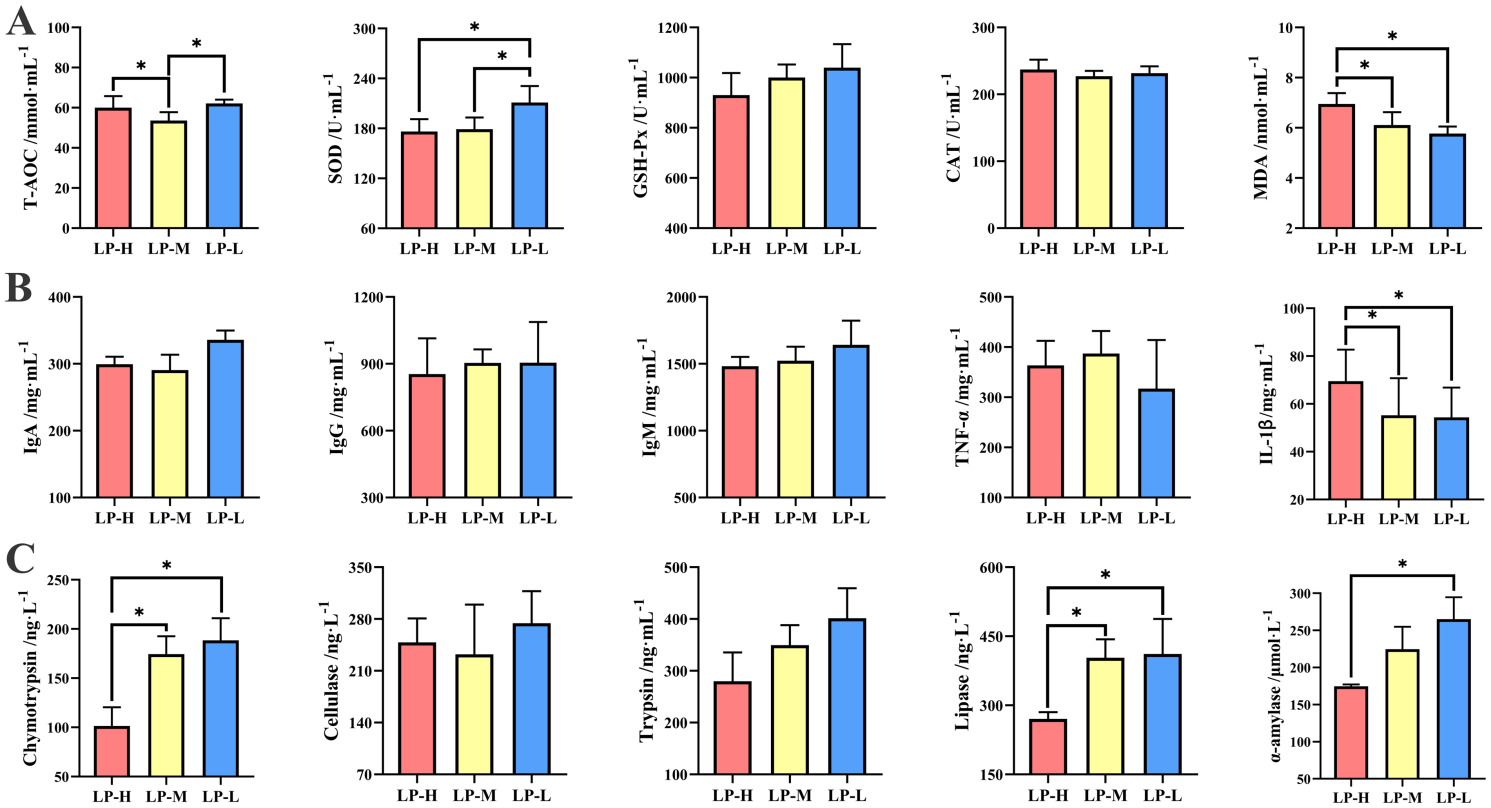
Effect of Lys/Met ratio on jejunal antioxidant capacity, immune status and digestive enzyme activity. **(A)** Antioxidant capacity including T-AOC, SOD, GSH-Px, CAT, and MDA. **(B)** Immune status including IgA, IgG, IgM, TNF-α, and IL-1β. **(C)** Digestive enzyme activity including α-amylase, chymotrypsin, cellulose, trypsin, and lipase. Data are presented as mean ± SD. *p < 0.05.

In terms of the antioxidant activity (Figure 2A), the LP-L group showed higher levels of SOD activity (*p* = 0.019) and T-AOC concentration (*p* = 0.044) compared to the LP-M and LP-H groups, whereas the MDA concentration was lower (*p* = 0.020). No significant difference in the activities of GSH-P (*p* = 0.309) and CAT (*p* = 0.575) among the three treatment groups was observed.

In terms of the immune levels (Figure 2B), a lower IL-1β (*p* = 0.049) concentration was observed in the LP-L group compared to the LP-H group. The concentrations of IgA (*p* = 0.456), IgG (*p* = 0.351), IgM (*p* = 0.871), and TNF-α (*p* = 0.235) of the sheep were not affected by the dietary Lys/Met ratios.

In terms of the digestive enzyme activities (Figure 2C), except for cellulose and trypsin, the activities of α-amylase (*p* = 0.034), chymotrypsin (*p* = 0.003), and lipase (*p* = 0.016) were higher in the LP-L group compared to the LP-H group.

### 3.3 Effect of the dietary Lys/Met ratios on the jejunal SCFA concentration

As shown in Table 2, the concentrations of acetic acid (*p* = 0.024) and propionic acid (*p* = 0.005) were significantly increased in the LP-L group compared to the LP-H group. There was no significant difference in the concentrations of isobutyric acid (*p* = 0.448), butyric acid (*p* = 0.887), isovaleric acid (*p* = 0.955), and pentanoic acid (*p* = 0.527) among the three treatment groups.

**Table 2:** Effect of the dietary Lys/Met ratio on the jejunal SCFA concentration.

Items	LP-H	LP-M	LP-L	*p*-value
Acetic acid/(mmol/mL)	30.23 ± 1.29^b^	31.81 ± 0.91^ab^	35.00 ± 0.98^a^	0.02
Propionic acid/(mmol/mL)	1.85 ± 0.08^b^	2.00 ± 0.05^ab^	2.33 ± 0.12^a^	0.01
Butyric acid/(mmol/mL)	1.83 ± 0.08	1.88 ± 0.07	1.84 ± 0.09	0.89
Isobutyric acid(mmol/mL)	0.39 ± 0.07	0.40 ± 0.06	0.40 ± 0.12	0.45
Pentanoic acid/(mmol/mL)	0.30 ± 0.03	0.31 ± 0.02	0.28 ± 0.01	0.53
Isovaleric acid/(mmol/mL)	0.66 ± 0.07	0.63 ± 0.08	0.64 ± 0.07	0.96

^a,b^Means with difference superscripts in the same row are significantly different (*p* < 0.05). Data are presented as mean ± SD.

### 3.4 Effect of the dietary Lys/Met ratios on the jejunal microbiota

**Figure 3:**
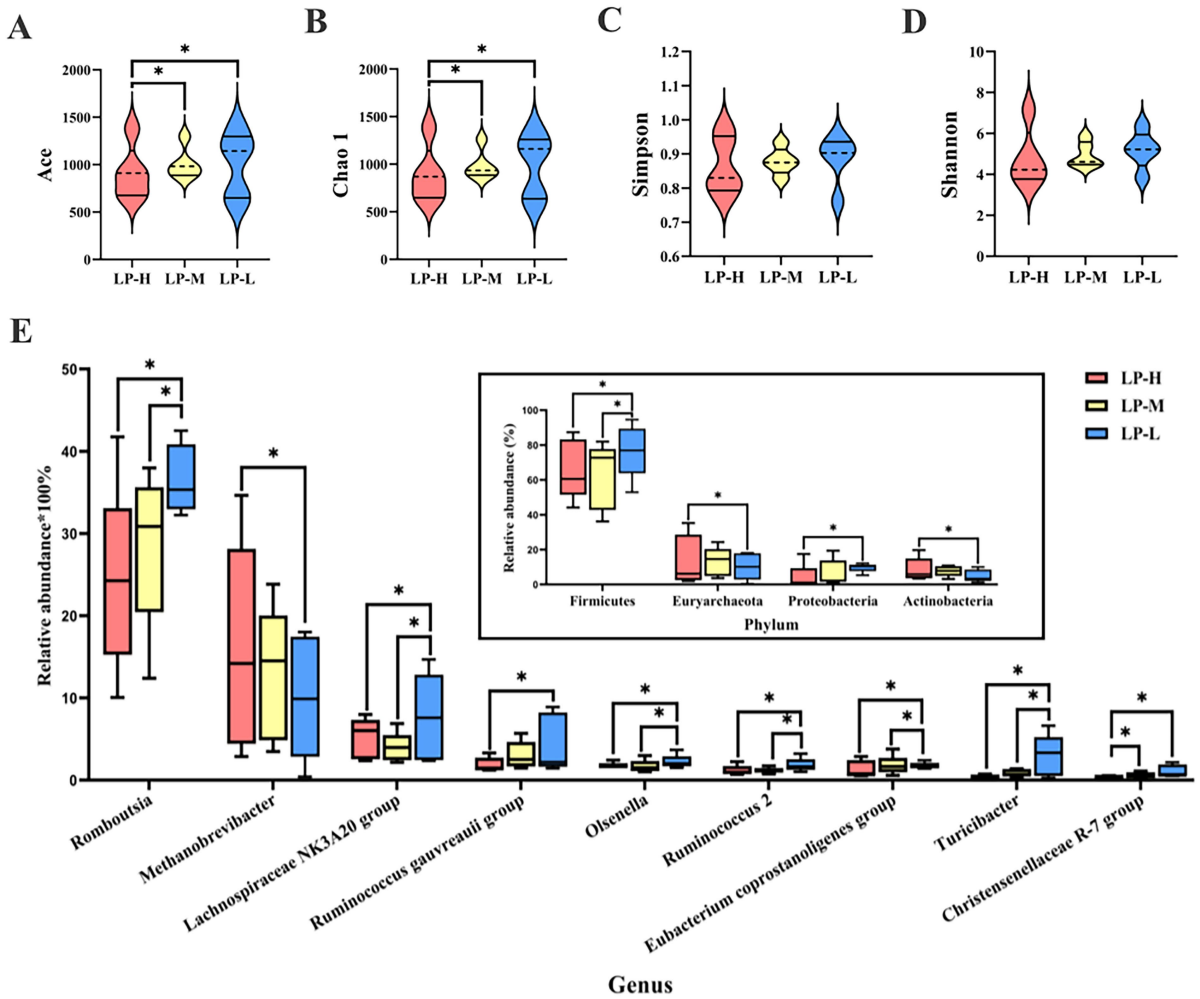
Effect of Lys/Met ratio on jejunal microbiome. Alpha diversity measured by the Ace **(A)**, Chao1 **(B)**, Simpson **(C)**, and Shannon **(D)** indexes. **(E)** Difference of microbiota at the genus level (only genera with relative abundance of >1.00%). Bar plot indicates that changes in phylum levels correspond to the targeted genera. *p < 0.05.

The microbiome diversity in the jejunal content with the different Lys/Met ratios was investigated using 16S rDNA gene sequencing. A total of 1,290,237 raw reads were generated, ranging from 77,538 to 95,217. The Venn diagram (Supplementary Figure S1) shows the common and unique OTUs: 977 common OTUs and 277 unique OTUs in the LP-L group, 499 unique OTUs in the LP-M group, and 358 unique OTUs in the LP-H group. When examining the community structure, both Chao1 and ACE richness was significantly increased in the LP-L group (*p* = 0.043 and *p* = 0.018) (Figures 3A,B), suggesting that species richness in the jejunum is connected to the ratio of dietary Lys/Met. No significant differences in the Shannon and Simpson indices among the treatment groups were observed (Figures 3C,D). The beta diversity of the microbiota was assessed using weighted UniFrac distance-based non-metric multidimensional scaling (NMDS). The results of the NMDS analysis showed distinct differences among the treatment groups (stress value = 0.1238).

The taxonomic analysis of the reads revealed that Firmicutes (68.50%), Euryarchaeota (12.34%), Actinobacteria (7.08%), Proteobacteria (6.81%), and Tenericutes (2.80%) were the dominant phyla in the jejunal content across all treatment groups (Figure 3E). Reducing the dietary Lys/Met ratio significantly increased the abundance of Tenericutes (*p* = 0.015) and Actinobacteria (*p* = 0.028), whereas it decreased the abundance of Firmicutes (*p* = 0.042) and Proteobacteria (*p* = 0.019). At the genus level, the predominant genera included *Methanobrevibacter* (13.21%), *Lachnospiraceae NK3A20* group (9.77%), *Paeniclostridium* (9.18%), *Gauvreauii* group (39.9%), and *Olsenella* (3.79%). The abundance of *Methanobrevibacter* (*p* = 0.029) was significantly down-regulated in the LP-L group. On the contrary, the abundance of *Olsenella* (*p* = 0.011), *Romboutsia* (*p* = 0.035), *Ruminococcus gauvreauii* group (*p* = 0.019), *Lachnospiraceae NK3A20* group (*p* = 0.048), *Ruminococcus 2* (*p* = 0.040), *Eubacterium coprostanoligenes* group (*p* = 0.005), *Turicibacter* (*p* = 0.038), and *Christensenellaceae R-7* group (*p* = 0.005) was significantly up-regulated.

The LEfSe analysis revealed relatively higher abundance of *Christensenellaceae R-7*, *Ruminococcaceae NK4A214* group, *Nodatum* group, and *Family XIII UCG-001* in the LP-L group, while the abundance of *Parvimonas* was enriched in the LP-H group (Supplementary Figure S2).

### 3.5 Correlation analysis of the phenotypes and microorganisms at the genus level of the jejunum

**Figure 4:**
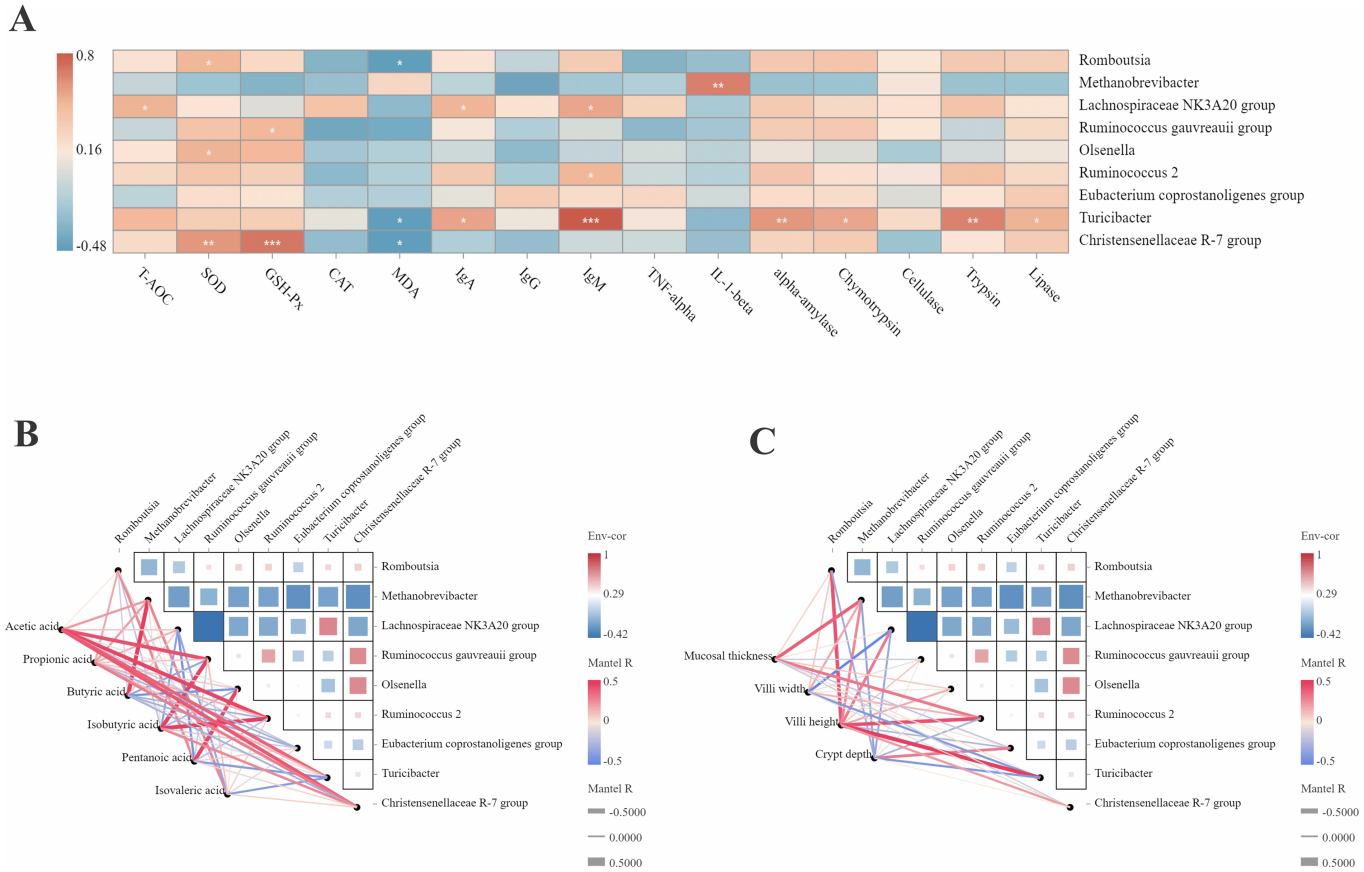
Correlation analysis between the differential microbiota with the phenotypic data. **(A)** Correlation analysis between the differential microbiota with the antioxidant capacity, immune status and digestive enzyme activity. *p < 0.05. **p < 0.01. ***p < 0.001. **(B)** Correlation analysis between the differential microbiota with the SCFAs concentration. **(C)** Correlation analysis between the differential microbiota with the jejunal morphologies. Red color represents a positive correlation, while blue color represents a negative correlation. The color intensity is proportional to the correlation values.

The correlation between the jejunal health and microorganisms was analyzed (Figure 4A). The abundance of *Romboutsia, Turicibacter,* and *Christensenellaceae R-7* group was negatively correlated with MDA (*r* < −0.5). The abundance of *Methanobrevibacter* was positively correlated with IL-1β (*r* > 0.5). The abundance of *Turicibacter* was positively correlated with IgM, α-amylase, chymotrypsin, and trypsin (*r* > 0.5). The abundance of *Christensenellaceae R-7* was positively correlated with SOD and GSH-P (*r* > 0.5).

The correlation analysis of the SCFA concentration and microorganisms in the jejunum is shown in Figure 4B. The abundance of *Methanobrevibacter* was positively correlated with butyric acid (*r* > 0.5). The abundance of *Ruminococcus gauvreauii* group was positively correlated with acetic acid and isobutyric acid (*r* > 0.5). The abundance of *Ruminococcus_2* was positively correlated with isobutyric acid (*r* > 0.5). The abundance of *Turicibacter* and *Christensenellaceae R-7* group was positively correlated with acetic acid (*r* > 0.5).

As shown in Figure 4C, the mucosal thickness was positively correlated with the abundance of *Methanobrevibacter* and *Ruminococcus_2* (*r* > 0.5). The villus width was negatively correlated with the abundance of the *Lachnospiraceae NK3A20* group (*r* < −0.5). The villus height was positively correlated with the abundance of *Romboutsia*, *Ruminococcus_2,* and *Turicibacter* (*r* > 0.5).

### 3.6 Effect of the dietary Lys/Met ratios on the metabolomic profiles

**Figure 5:**
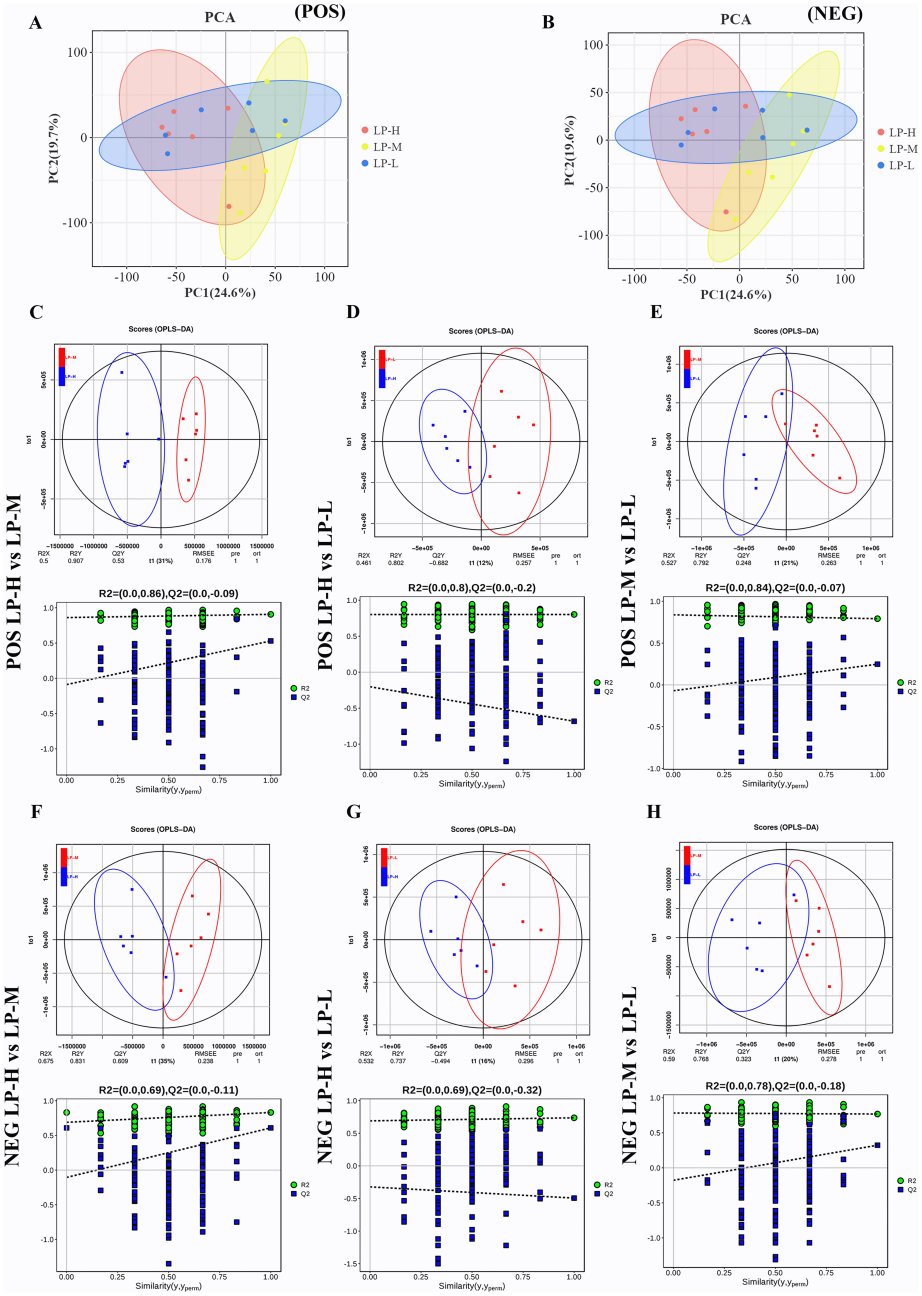
Principal coordinate analysis (PCoA) and orthogonal partial least squares discriminant analysis (OPLS-DA) scores of jejunal metabolism. **(A)** PCoA of positive ion mode. **(B)** PCoA of negative ion mode. **(C)** OPLS-DA of LP-M vs LP-L in positive ion mode. **(D)** OPLS-DA of LP-M vs LP-L in negative ion mode. **(E)** OPLS-DA of LP-H vs LP-L in positive ion mode. **(F)** OPLS-DA of LP-H vs LP-L in negative ion mode. **(G)** OPLS-DA of LP-M vs LP-H in positive ion mode. **(H)** OPLS-DA of LP-M vs LP-H in negative ion mode.

A total of 2,377 metabolites were identified in the jejunal metabolome using positive and negative ion modes. The Principal component analysis (PCA) (Figures 5A,B) and orthogonal partial least squares discriminant analysis (OPLS-DA) models (Figures 5C–H) revealed clear distinctions among the low protein-low methionine (LP-L), low protein-medium methionine (LP-M), and low protein-high methionine (LP-H) groups. The OPLS-DA models showed good fit and predictability, with *R*^2^ and *Q*^2^ values ranging from 0.391 to 0.999 and −0.510 to 0.675, respectively. The permutation tests confirmed the effectiveness of the models, with R2 values ranging from 0.727 to 0.968.

Based on VIP >1 and a *p*-value < 0.05, 321 metabolites differed significantly among the treatment groups, with 187 and 134 detected in the positive and negative ion modes, respectively (Supplementary Table S2). These metabolites were classified into various categories, including lipids and lipid-like molecules (24.92%), organic acids and derivatives (23.05%), and benzenoids (14.01%). The pathway enrichment analysis revealed nine significantly enriched metabolic pathways (Supplementary Figure S3), including glycine, serine, and threonine metabolism; vitamin digestion and absorption; and protein digestion and absorption. Nine differential metabolites were derived from common metabolic processes, such as beta-alanine, pantothenate, and cysteine.

### 3.7 Correlation analysis of the differential metabolites and phenotypes of the jejunum

**Figure 6:**
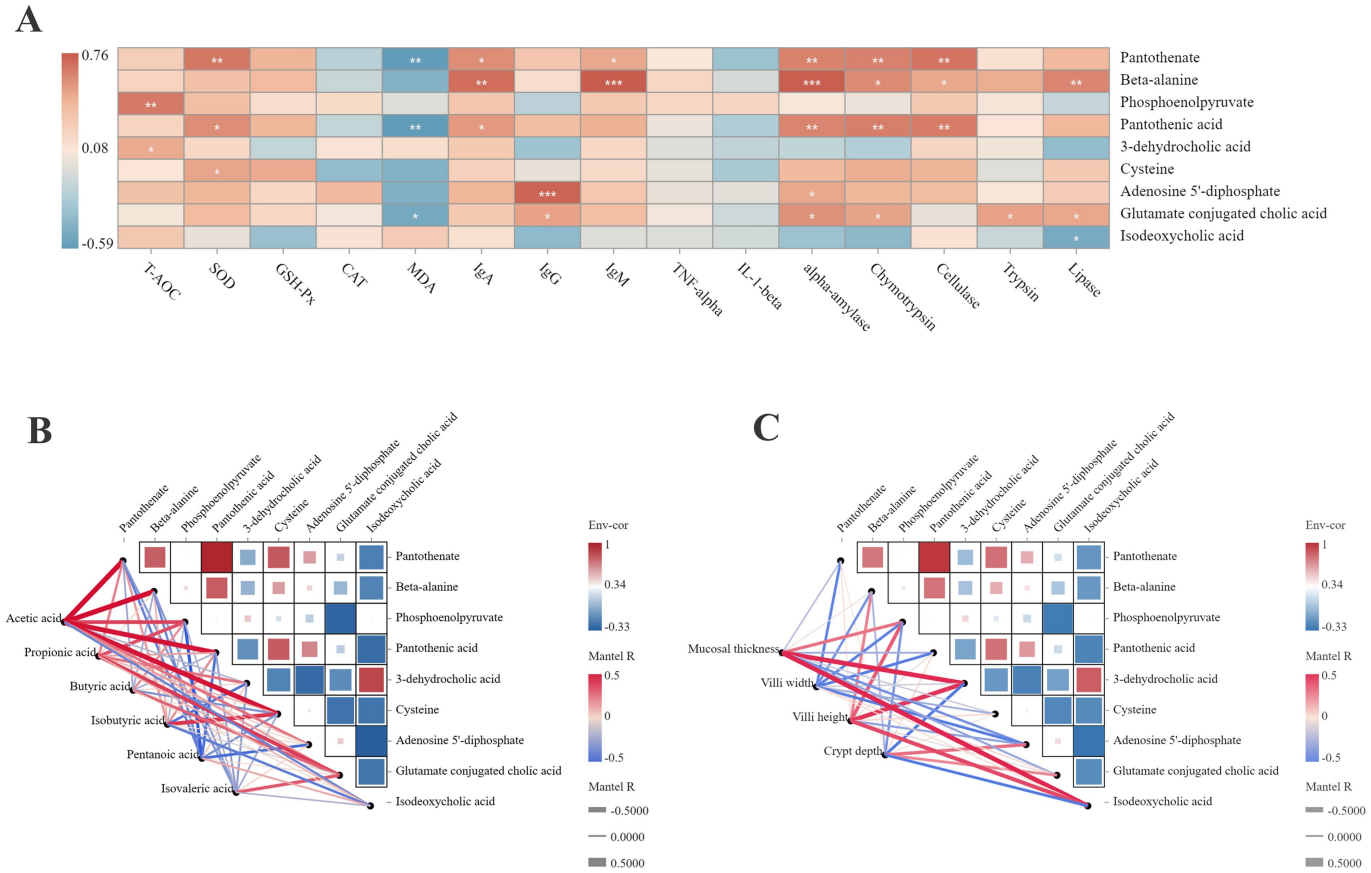
Correlation analysis between the differential metabolites with the phenotypic data. **(A)** Correlation analysis between the differential metabolites with the antioxidant capacity, immune status and digestive enzyme activity. *p < 0.05. **p < 0.01. ***p < 0.001. **(B)** Correlation analysis between the differential metabolites with the SCFAs concentration. **(C)** Correlation analysis between the differential metabolites with the jejunal morphologies. Red color represents a positive correlation, while blue color represents a negative correlation. The color intensity is proportional to the correlation values.

Figure 6A shows the correlation network between the differential metabolites and jejunal health. Pantothenate was positively correlated with SOD, α-amylase, chymotrypsin, and cellulase (*r* > 0.5). β-alanine was positively correlated with IgA, IgM, α-amylase, and lipase (*r* > 0.5). Phosphoenolpyruvate was positively correlated with T-AOC (*r* > 0.5). Pantothenic acid was positively correlated with α-amylase, chymotrypsin, and cellulase (*r* > 0.5). Adenosine 5′-diphosphate was positively correlated with IgG (*r* > 0.5). Pantothenate, pantothenic acid, and glutamate-conjugated cholic acid were negatively correlated with MDA (*r* < −0.5).

The correlation between the SCFA concentration and differential metabolites (Figure 6B) showed that acetic acid was positively correlated with pantothenate, β-alanine, pantothenic acid, cysteine, and glutamate-conjugated cholic acid (*r* > 0.5). Propionic acid was positively correlated with phosphoenolpyruvate (*r* > 0.5). Isobutyric acid was positively correlated with cysteine (*r* > 0.5). Pentanoic acid was negatively correlated with phosphoenolpyruvate and pantothenic acid (*r* < −0.5).

Figure 6C illustrates the correlation between the differential metabolites and jejunal morphology. The mucosal thickness was positively correlated with phosphoenolpyruvate, 3-dehydrocholic acid, and isodeoxycholic acid (*r* > 0.5). The villus height was positively correlated with β-alanine, phosphoenolpyruvate, 3-dehydrocholic acid, and isodeoxycholic acid (*r* > 0.5). The villus width was negatively correlated with pantothenate, phosphoenolpyruvate, and pantothenic acid (*r* < −0.5).

### 3.8 Effect of the Lys/Met ratios on the expression of the barrier-related genes in the jejunum

**Figure 7:**
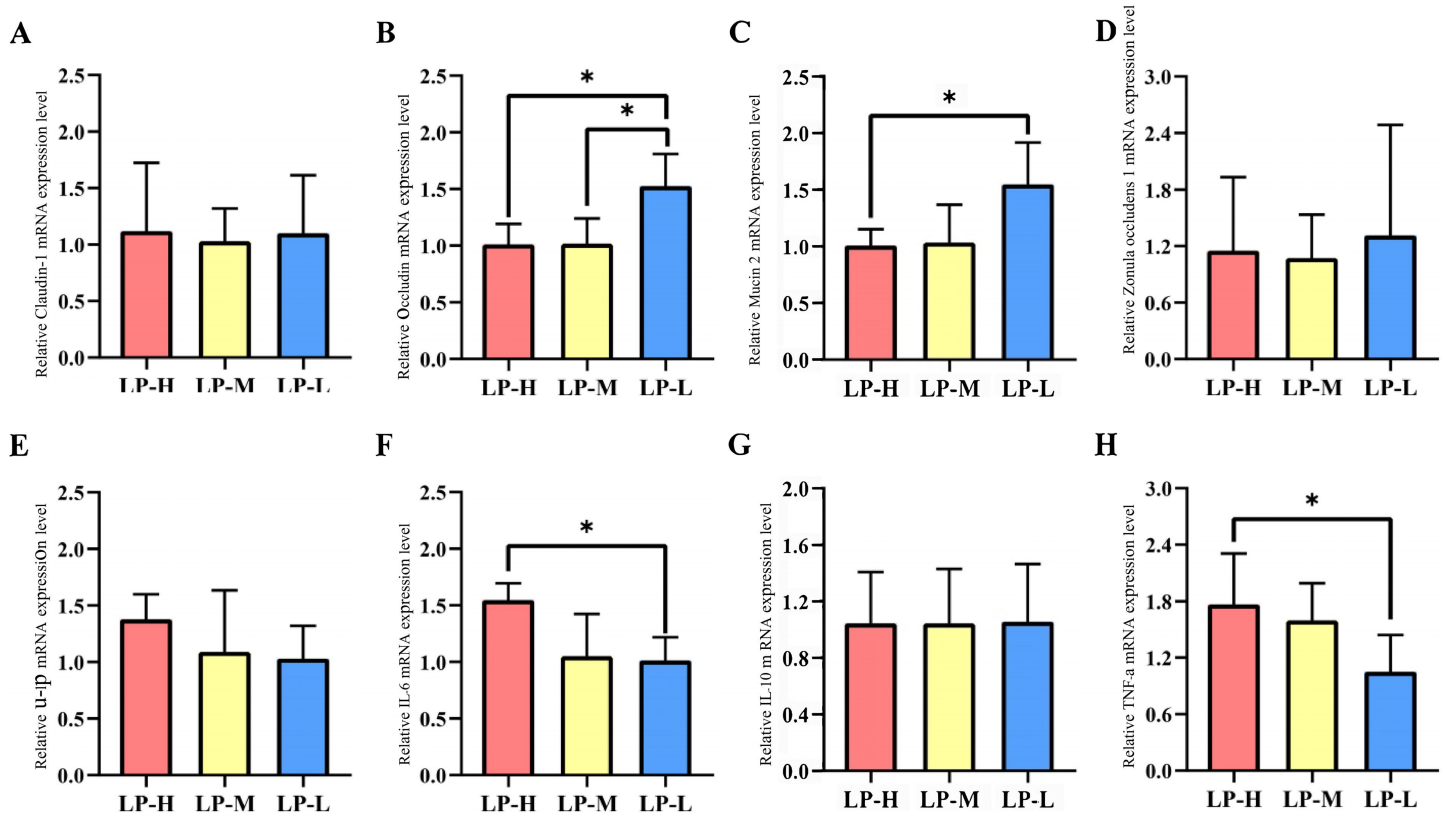
Effect of Lys/Met ratio on barrier-related genes expression. **(A)** Claudin-1. **(B)** Occludin. **(C)** Muc-2. **(D)** ZO-1. **(E)** IL-1β. **(F)** IL-6. **(G)** IL-10. **(H)** TNF-α. Data are presented as mean ± SD. *p < 0.05.

The relative mRNA expression in the jejunum tissue was analyzed among the three Lys/Met ratios (Figure 7). Occludin and Muc-2 were up-regulated in the LP-L group (*p* < 0.05), while IL-6 and TNF-α were down-regulated (*p* < 0.05).

## 4 Discussion

Dietary EAA supplementation could affect growth performance, the humoral and cellular immune system, and the microbiota community (Peng et al., 2016; Fan et al., 2017). A moderate reduction in dietary protein levels enriched with nutritionally indispensable AAs is beneficial to host immunity, intestinal microbiota, gut health, and N utilization efficiency (Wang et al., 2019). In the current intensive feeding system, low-protein diets supplemented with EAAs are widely used to reduce costs and N emissions and improve efficiency (Gebeyew et al., 2021). Dietary supplementation with Met and Lys plays a key role in promoting growth, maintaining intestinal integrity, enhancing immunometabolic status, and regulating the immune system and intestinal microbiota (Zhou et al., 2016; Elolimy et al., 2019). In the current study, we aimed to investigate the effects of different Lys/Met ratios on jejunal morphology, digestive ability, antioxidant capacity, immune status, and microbiota in Tibetan sheep fed a low-protein diet.

Jejunal morphology, including villi, mucosa, and crypts, is responsible for lipid digestion and absorption in enterocytes (Lema et al., 2020). This morphology interacts with insulin resistance (Castagneto-Gissey et al., 2020), immune response (He Y. et al., 2023), and antioxidant capacity (Xu et al., 2022). As previously mentioned, villus height and crypt depth were greater in the duodenum of pigs fed diets supplemented with DL-methionine hydroxy analog (Namted et al., 2020). Similarly, an increasing trend in villus width was observed with higher dietary DL-Met supplementation (Morales et al., 2023). Injecting DL-Met into chick embryos during late-term embryonic development improved intestinal development, especially in villus width (Lugata et al., 2024). The data from the present study are consistent with these findings, as the dietary Lys/Met ratios significantly influenced the villus height in the jejunum. However, it is well documented that an inappropriate essential AA ratio or essential AA deficiency could impair intestinal morphology. A diet with a Met:SAA ratio of 0.61 suppressed villus height and decreased the VH:CD ratio in the ileum of weaned piglets (Bai et al., 2020). On the other hand, reducing dietary Met supplementation inhibited the abundance of mRNA coding for the neutral AA transporter B0 in the ileum, resulting in the suppression of villus height and crypt depth in heat-stressed pigs (Morales et al., 2023). In the present study, the dietary Lys/Met ratios significantly influenced the villus height in the jejunum, suggesting that a higher Lys/Met ratio may promote the development of the jejunum, especially villus growth, in Tibetan sheep.

Common oxidative biomarkers, including T-AOC, SOD, CAT, GPX, and GSH-Px, were studied as responses to dietary Met supplementation in broilers (Chang et al., 2022), cattle (Mesgaran et al., 2022), sheep (Liu R. et al., 2021), and piglets (Zeitz et al., 2019). Dietary Me restriction directly impacted the redox state of lambs by activating the Nrf2 signaling pathway (Liu R. et al., 2021). By modulating the expression of SOD and CAT, liver damage induced by LPS challenge was alleviated in lambs fed Met-treated superoxide diets (Liu et al., 2022). For these different biomarkers, SOD was found to eliminate superoxide free radicals and protect cellular normal physiological conditions, thereby maintaining internal dynamic balance (Fan et al., 2021). However, MDA was found to exert cytotoxic effects since it oxidizes DNA, proteins, and lipids (Bernabucci et al., 2002). In the current study, we observed that the concentrations of T-AOC, SOD, and MDA in the jejunal content exhibited significant differences among the three treatment groups, indicating that the jejunal antioxidant capacity increased with the rise in the Met content in the diet. One possible explanation is that dietary Met supplementation contributes to oxidation resistance by producing antioxidants (such as precursors of cysteine and glutathione) (Santana et al., 2021).

In a previous study, ewe supplementation with Met promoted the mRNA expression of *JunD* while inhibiting the mRNA expression of *IL-10* and *HO-1* in monocytes (Tsiplakou et al., 2020). Under long-term environmental heat stress, dietary Met supplementation reduced the concentrations of haptoglobin and IL-1B, thereby improving the immune status of dairy cows (Mesgaran et al., 2022). Moreover, both *TNF-*α and *IFN-γ* mRNA expressions were suppressed after Met supplementation in the jejunum of Cherry Valley ducks (Chang et al., 2022). In this study, a significant reduction in IL-1β in the jejunum was observed in the ewes fed the Lys/Met ratio of 1:1. These outcomes are consistent with previous studies, which have provided evidence that dietary Met alleviates inflammatory responses in lipopolysaccharide-challenged animals by decreasing IL-1β, IL-6, and TNF-α contents (Pang et al., 2023). In this study, a significant reduction in IL-1β in the jejunum was observed in the ewes of the LP-L group with the Lys/Met ratio of 1:1, suggesting that a higher Met level in the diet alleviates the inflammatory response in Tibetan sheep.

Based on the observed phenotypic traits, varying Lys/Met ratios not only significantly affect short-term health markers but also impact long-term productivity and overall animal health. Over the short term, total nitrogen excretion per metabolic body weight tended to be lower during the early period when the limiting amino acid was supplemented in Holstein steers (Kamiya et al., 2021). The maternal plasma hydrogen sulfide (H_2_S) concentration increased quadratically with the dietary Met/Lys ratio at embryonic days (E) E90 and E114, which was positively correlated with the number of piglets born alive and litter weight (Peng et al., 2020). Over the long term, providing a low-protein diet supplemented with Lys and Met increases hepatic nitrogen utilization and the expression of genes associated with nitrogen and urine metabolism. Therefore, we hypothesized that dietary Lys-to-Met ratios could first modulate nitrogen metabolism and then alter SCFA concentrations in the intestinal tract, thereby improving morphology, antioxidant defense, immune responses, and jejunal barrier function.

The intestinal microbiome is crucial for host health, influencing nutrient metabolism, immune response, and disease occurrence (Wang et al., 2020). Accumulating evidence has revealed that supplementation with AAs, especially Lys and Met, affected the composition of gastrointestinal microbiota (Yu et al., 2024). Consistent with these studies, our results showed that both Chao1 and ACE indices exhibited significant differences, indicating that the species richness of the jejunal microbiota is connected to the ratio of dietary Lys/Met. In a previous study, changes in the abundance of several bacterial taxa, including increases in *Christensenellaceae_R-7_group* and decreases in *Prevotellaceae _YAB2003_group* and *Succinivibrio*, were observed when Holstein bulls were fed a diet supplemented with 9 g/d Met (Zou et al., 2023). The abundance of genes involved in VFA synthesis (*Acinetobacter, Lactococcus, Microbacterium, Chryseobacterium,* and *Klebsiella*) increased in the rumen of lactating buffaloes fed Met alone or in combination, whereas the abundance of *Prevotell*, *Prevotellaceae_UCG-001*, *Succiniclasticum, Prevotellaceae_UCG-003,* and *Christensenellaceae_R-7_group* decreased (Guo et al., 2023). In the present study, Firmicutes (68.50%) was the main bacterial phyla and *Methanobrevibacter* (13.21%) was the main genus, which is in agreement with earlier reports on jejunal bacteria in sheep (Ye et al., 2022; Zhong et al., 2022). Moreover, increasing the dietary Lys/Met ratio significantly decreased the abundance of Tenericutes and *Actinobacteria* while increasing the abundance of Firmicutes and Proteobacteria. As a core bacterial component of the gastrointestinal tract, the phylum Firmicutes contains many fiber-degrading bacteria (e.g., *Ruminococcus*, *Butyvibrio*, and *Eubacterium*), which can degrade dietary fiber to produce VFAs (Chen X. et al., 2022). By maintaining an anaerobic environment, the phylum Proteobacteria is mainly involved in degrading structural carbohydrates and is associated with fermentation parameters (Moraïs and Mizrahi, 2019). Moreover, the increased abundance of Firmicutes, known for their involvement in fiber fermentation and SCFA production, might be beneficial for enhancing the host’s ability to extract energy from dietary fibers. This could potentially improve overall metabolic efficiency.

In grazing lactating yaks, an increasing trend was observed in the relative abundance of *Christensenellaceae_R-7_group* and *Ruminococcus_1* when fed diets supplemented with Met (Liu et al., 2021a). The addition of Met to the diet of Holstein Bulls significantly increased the relative abundance of *Christensenellaceae_R-7_group* (Zou et al., 2023). These findings are in line with those of the current study, where the abundance of *Romboutsia*, *Ruminococcus gauvreauii* group, *Lachnospiraceae NK3A20* group, *Ruminococcus 2, Eubacterium coprostanoligenes* group, and *Christensenellaceae R-7* group was significantly increased as the dominant genera when the Tibetan sheep were fed a low-Lys/Met (1:1) diet. Interestingly, most of these differential bacteria including *Ruminococcus gauvreauii* group, *Ruminococcus 2,* and *Christensenellaceae R-7* group are strongly correlated with SCFA concentrations. In detail, the *Ruminococcus gauvreauii* group participates in the degradation of glycogen by converting glucose into acetic acid (Domingo et al., 2008). *Ruminococcus 2* belongs to the genus of *Ruminococcus,* which acts mainly on the degradation of polysaccharides and produces SCFAs (Zhang et al., 2017). The *Christensenellaceae R-7* group has been identified as a biomarker contributing to the combined effect of host genetics and SCFAs in the gastrointestinal tract (He Z. et al., 2023). Therefore, we speculated that a low-Lys/Met (1:1) diet could promote the SCFA concentration in the jejunum by altering the abundance of SCFA-related bacteria, thereby contributing to the fermentation of cellulose, hemicellulose, and fiber into end products that are utilized by the host (Wu et al., 2022). These changes have a significant effect on host maintenance, growth, and health.

To confirm this speculation, the SCFA concentrations in the jejunal contents were measured using gas chromatography in our study. As the dietary Lys/Met ratio decreased, the concentrations of acetic acid and propionic acid increased in the Tibetan sheep. These results are consistent with a previous study on Hu sheep, which reported that dietary 2-hydroxy 4-(methylthio)-butanoic acid (HMBi) altered the rumen microbiota and metabolites, resulting in higher concentrations of total volatile fatty acid, acetate, and propionate (Li S. et al., 2022). In addition, a positive correlation between the differential bacteria (e.g., *Ruminococcus gauvreauii* group*, Ruminococcus 2,* and *Christensenellaceae R-7* group) and SCFA concentrations (e.g., acetic acid and propionic acid) was observed in the current study. It also supported the speculation that the dietary Lys/Met ratio alters the abundance of SCFA-related bacteria, thereby contributing to the production of SCFAs. A possible explanation for this is that dietary Met can accelerate the synthesis of microbial crude protein (MCP) in the jejunum, thereby increasing the abundance of SCFA-related bacteria (Li Y. et al., 2022).

Accumulating evidence has suggested that the gut microbiome interacts with the host by producing a mass of metabolome, thereby modulating intestinal barrier function and immune response (Ghosh et al., 2021). Compared to genomics or proteomics, metabolomics can comprehensively explain the mechanism of intestinal phenotypic changes (Ke et al., 2015). Previous research identified that secondary bile acids and sphingosine metabolites of the jejunum were affected by the dietary methionine source in pigs (Schermuly et al., 2022). Our results showed that a total of nine differential metabolites were derived from common metabolic processes, including beta-alanine, pantothenate, pantothenic acid, phosphoenolpyruvate, cysteine, adenosine 5′-diphosphate, isodeoxycholic acid, glutamate conjugated cholic acid, and 3-dehydrocholic acid. The pathway enrichment analysis revealed that these differential metabolites were mainly involved in pantothenate and CoA biosynthesis and the TCA cycle. Pantothenate, also referred to as vitamin B5, is the universal precursor of CoA, which plays an important role in metabolic regulation (Jonczyk et al., 2007). As an essential cofactor, CoA and its derivatives are mainly involved in the metabolism of pyruvate, which can stimulate the TCA cycle and provide energy requirements for the host (Zhang S. et al., 2024). By oxidizing the acetyl group of acetyl-CoA, the mitochondrial TCA cycle generates reducing equivalents and modulates the synthesis of ATP (Sanchez-Gimenez et al., 2023). In addition, previous studies have shown that pantothenate and CoA biosynthesis and the TCA cycle are key signaling pathways closely related to SCFAs (Chen K. et al., 2022; Kim et al., 2022). In the current study, acetic acid was positively correlated with pantothenate, β-alanine, pantothenic acid, cysteine, and glutamate-conjugated cholic acid. Propionic acid was positively correlated with phosphoenolpyruvate. Isobutyric acid was positively correlated with cysteine. Pentanoic acid was negatively correlated with phosphoenolpyruvate and pantothenic acid. These findings suggest that dietary Lys and Met are involved in the regulation of SCFAs in Tibetan sheep by modulating the jejunal metabolome. However, further investigations using functional analysis to determine the hierarchy of microbiome and metabolite alterations in relation to dietary Lys/Met-induced effects on the digestive system of Tibetan sheep are warranted.

The intestinal physical barrier serves as the first line of defense against antigens or harmful microorganisms and consists of intestinal epithelial cells, tight junctions, and intestinal mucosa (Cui et al., 2019). The tight junctions, including Claudin-1, Occludin, Mucin 2, and Zonula occludens 1, are correlated with the polarity of epithelial cells and cellular permeability (Xie et al., 2011). Claudin-1 is predominantly expressed in the intestinal epithelium and is known for its barrier-forming ability, playing an important role in tight junction integrity. By reducing the uptake of toxins during pathogenic bacterial infections, Occludin contributes to the tightness of intestinal epithelial cells (Phillippi et al., 2022). As a marker of gut health in mammals, Mucin 2 covers the intestinal epithelial surface and protects against inflammatory diseases (Hui et al., 2020). Zonula occludens 1 belongs to the membrane-associated guanylate kinase protein family, allowing interaction with cell adhesion proteins and cytoskeletal components (Maytorena-Verdugo et al., 2022). In the present study, the relative expressions of Occludin and Mucin 2 in the jejunum were up-regulated when the Tibetan sheep were fed a low-Lys/Met (1:1) diet, indicating that the dietary Lys/Met ratio could influence the jejunal physical barrier and tight junctions. It is possible that dietary Lys/Met supplementation alters the jejunal SCFA concentration, thereby affecting the jejunal physical barrier by regulating the expression of barrier-related genes (Diao et al., 2019).

Previously, several studies have reported that supplementing appropriate amino acids in diets alleviates heat stress-induced metabolic diseases in cows (Guo et al., 2018; Koch et al., 2021). However, excessive amino acid supplementation results in digestive tract disturbances, thereby negatively affecting growth performance. The results of the present study suggest that supplementation of Lys:Met at a 1:1 ratio in an LP diet (10% CP) is a practical strategy to improve jejunal function in Tibetan sheep, indicating that balancing the dietary Lys:Met ratio could promote nutrient absorption and effectively reduce CP intake.

## 5 Conclusion

**Figure 8:**
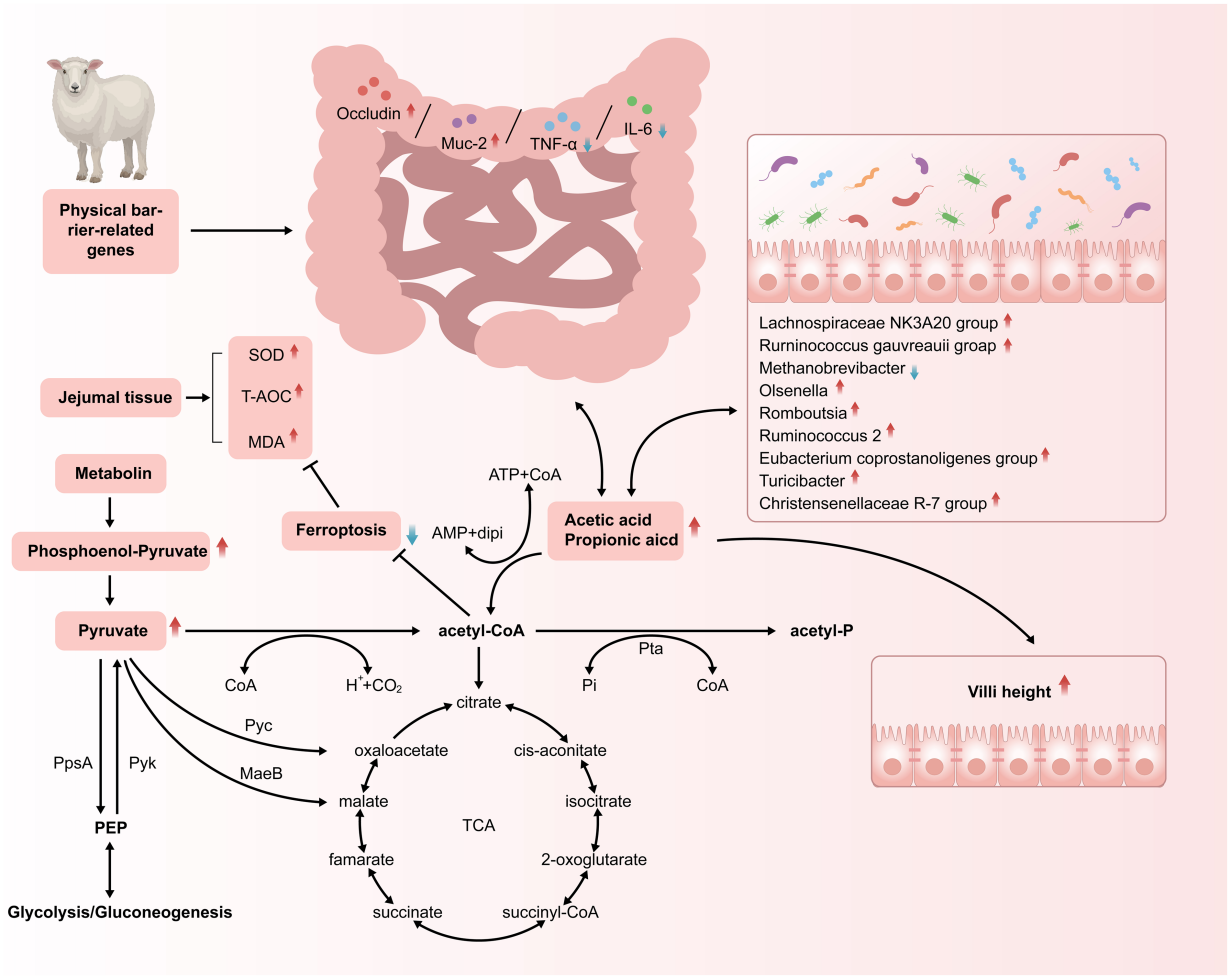
Schematic illustration demonstrating how feeding different Lysine-to-Methionine ratio in a low protein diet to Tibetan sheep altered the SCFAs profiles, villus morphology, antioxidant capacity and immune status of jejunum.

The dietary Met/Lys ratio significantly influenced the concentration of the jejunal SCFAs by modulating the microbial community, including *Romboutsia*, the *Ruminococcus gauvreauii* group, the *Lachnospiraceae NK3A20* group, *Ruminococcus 2*, and *Christensenellaceae R-7*. It also regulated the metabolic pathways such as pantothenate, pantothenic acid, phosphoenolpyruvate, isodeoxycholic acid, and 3-dehydrocholic acid, contributing to enhanced jejunal barrier function. The proposed mechanism of the dietary Met/Lys ratio’s effects on the SCFA concentration, villus development, immune status, antioxidant activity, microbiota composition, and metabolism is illustrated in Figure 8. These findings provide a robust theoretical basis for the application of Lys/Met diet supplementation in the nutritional management of Tibetan sheep, particularly in reducing dietary crude protein levels while maintaining health and performance. Further studies are needed to determine the optimal dietary protein content and the appropriate amounts of supplemental Lys and Met at different production stages of Tibetan sheep.

## Data Availability

The datasets presented in this study can be found in online repositories. The names of the repository/repositories and accession number(s) can be found below: NCBI SRA (accession: PRJNA1116617). China National Center for Bioinformation (accession: OMIX008918 https://www.cncb.ac.cn/).
