# Supplementary material for: Different lysine-to-methionine ratios in a low-protein diet affect the microbiome and metabolome, influencing the jejunal barrier function in Tibetan sheep
Source: Front Microbiol. 2025 Feb 12;16:1441143. doi: 10.3389/fmicb.2025.1441143 (PMC11861081; doi:10.3389/fmicb.2025.1441143)
Supplement: Supplementary file 1 [file Table_1.docx]

**Table 1S Primers used in qRT-PCR**

| Name | Primer sequence (5’-3’) | Tm (℃) | Product length |
| --- | --- | --- | --- |
| Claudin-1 | F-CCTGCTGTGCTGCTCCTGTC  R-GAAGGTGCTGGCTTGGGATAGG | 61.6  61.4 | 75bp |
| Occludin | F-CGAGAAGCGACCGTATCCAGAG  R-TCCAAGTTACCACTGCTGCTGTAG | 61.4  59.6 | 129bp |
| Mucin 2 | F-ACGACTCCTACGCCCTCCTG  R-ACGCTGCCATCCGACTTGAAG | 61.6  59.5 | 130bp |
| Zonula occludens 1 | F-GGGCAAGTTAAAGATGGTGGTTCAG  R-GAGGCGTCAGCAGAGTGGATG | 59.6  61.5 | 93bp |
| TNF-α | F-ACGGCGTGGAGCTGAAAGAC  R-CTGAAGAGGACCTGCGAGTAGATG | 59.5  61.3 | 79bp |
| IL-6 | F-TCTAATAACCACTCCAGCCACACAC  R-TTGCGTTCTTTACCCACTCGTTTG | 59.6  57.9 | 77bp |
| IL-1β | F-GGCAGGCAGTGTCGGTCATC  R-CCTCAGGTCATCATCACGGAAGAC | 61.6  61.3 | 83bp |
| IL-10 | F-AATGAAGGACCAACTGAACAGCATG  R-TCCGACAAGGCTTGGCAACC | 57.9  59.5 | 87bp |
| β-Actin | F-AGCAAGCGTGGCATCCTAACC  R-ATCTTCTCCATGTCGTCCCAGTTG | 59.5  59.6 | 77bp |

**Table 2S. Significant differential metabolites in positive and negative ion modes**

| **Name** | **ANOVA *P-*value** | **adduct** |
| --- | --- | --- |
| Norfenfluramine | 3.00909E-05 | [M+H]+ |
| 1-o-hexadecyl-2-o-acetyl-sn-glyceryl-3-phosphoryl(n,n,n-trimethyl)hexanolamine | 4.29013E-05 | [M+K]+ |
| Batyl alcohol | 0.000582268 | [M+Na]+ |
| Irbesartan | 0.000880808 | [M+H]+ |
| Ferrioxamine | 0.00138377 | [M+H]+ |
| Diosgenin | 0.001398094 | [M+H-H2O]+ |
| Dihydrokaempferol | 0.001488745 | [M+H-H2O]+ |
| Diferuloyl putrescine | 0.001493204 | [M+H]+ |
| [(2e,6z,10z)-7,11-bis(acetyloxymethyl)-3,15-dimethylhexadeca-2,6,10,14-tetraenyl] acetate | 0.001626058 | [M+H]+ |
| N.epsilon.-methyl-l-lysine | 0.001767715 | [M+H-NH3]+ |
| Methanone, (1-butyl-1h-indol-3-yl)(4-methoxy-1-naphthalenyl)- | 0.001788863 | [M+H-C11H10O]+ |
| 4'-o-.beta.-d-glucosyl-5-o-methylvisamminol | 0.001963239 | [M+H]+ |
| Milrinone | 0.00204554 | [M+H]+ |
| Tebutam | 0.002183256 | [M+H]+ |
| Benzamide | 0.002213871 | [M+H]+ |
| Plnh | 0.002769105 | [M+H+K]2+ |
| Trp-Arg | 0.002775729 | (M-H+2Na)+ |
| Gln-pro | 0.003160472 | [M+H]+ |
| 1-docosahexaenoyl-2-stearoyl-sn-glycero-3-phosphocholine | 0.003189232 | [M-H+2Na]+ |
| 2-aminophenol | 0.003274483 | [M+H-H2O]+ |
| Leukotriene f4 | 0.003384102 | [M+H]+ |
| (r)-4-((5s,8r,9s,10s,13r,14s,17r)-10,13-dimethyl-3,7-dioxohexadecahydro-1h-cyclopenta[a]phenanthren-17-yl)pentanoic acid | 0.003875594 | [M+H]+ |
| Ergothioneine | 0.003961231 | [M+H]+ |
| 2-hydroxy-2-methylbutyric acid | 0.003990996 | [M+H-H2O]+ |
| Eremanthin | 0.00468408 | [M+Na]+ |
| Lys-Ile-Lys | 0.004694185 | [M+H]+ |
| Met-Met-Arg | 0.004894035 | [M+H]+ |
| 4-hydroxy-l-isoleucine | 0.005437798 | [M+H]+ |
| Echimidine | 0.005966177 | [M+H]+ |
| Eriodictyol 7-o-neohesperidoside | 0.006251975 | [M+H-C6H10O5]+ |
| Adenosine 3'-monophosphate | 0.006504385 | [M+H]+ |
| 3.alpha.-hydroxy-7-oxo-5.beta.-cholanic acid | 0.006527415 | [M+H-3H2O]+ |
| Sn-glycerol-3-phosphoethanolamine | 0.006631468 | [M+Na]+ |
| (s)-10-hydroxycamptothecin | 0.006819997 | [M+H-CO2]+ |
| .gamma.-undecalactone | 0.006990734 | [2M+H]+ |
| 7.alpha.,12.alpha.-dihydroxycholest-4-en-3-one | 0.007127799 | [M+H]+ |
| Eprosartan | 0.007214893 | [M+H]+ |
| Beclomethasone | 0.007248742 | [M+H]+ |
| 3-amino-2,3-dihydrobenzoic acid | 0.007576693 | [M+H]+ |
| Fludrocortisone | 0.007666807 | [M+H]+ |
| 7-ethoxyresorufin | 0.007846696 | [M+H]+ |
| Mifepristone | 0.007955672 | [M+H]+ |
| 6-benzylaminopurine | 0.008962898 | [M+H]+ |
| DL-valine | 0.009078414 | [M+H]+ |
| 1-o-hexadecyl-2-o-(5z,8z,11z,14z,17z-eicosapentaenoyl)-sn-glyceryl-3-phosphorylcholine | 0.009102892 | [M+H]+ |
| Pyridoxamine | 0.009165998 | [M+H]+ |
| Hecogenin | 0.009195312 | [M+H]+ |
| 5,9-dihydroxy-5,7,7-trimethyl-4,5a,6,8,8a,9-hexahydro-1h-azuleno[5,6-c]furan-3-one | 0.009414055 | [M+K]+ |
| 6-methoxybenzoxazolin-2(3h)-one | 0.009453421 | [M+H]+ |
| Bufotalin | 0.009517601 | [M+H]+ |
| Benzamide, n-[2-[[(3r)-1-[trans-4-hydroxy-4-(6-methoxy-3-pyridinyl)cyclohexyl]-3-pyrrolidinyl]amino]-2-oxoethyl]-3-(trifluoromethyl)- | 0.009624044 | [M+H]+ |
| Flufenamic acid | 0.00970821 | [M+H-H2O]+ |
| Nudifloramide | 0.009985893 | [M+H]+ |
| Dl-threonine methyl ester | 0.010639385 | [M+H]+ |
| D-xylose | 0.010678936 | [M+H]+ |
| Prostaglandin g2 | 0.010747136 | [M+Na]+ |
| Leu-Gln-Lys | 0.010822707 | [M+H]+ |
| Norcodeine | 0.011054921 | [M+H]+ |
| Isoproterenol | 0.011323839 | [M+H-H2O]+ |
| Crotamiton | 0.011633355 | [M+H]+ |
| [(2r,3r,4s,5s,6r)-3-acetyloxy-2-(acetyloxymethyl)-5-hexanoyloxy-6-[(2s,3r)-2,3,4-trihydroxybutoxy]oxan-4-yl] 14-hydroxytetradecanoate | 0.011886555 | [M+Na]+ |
| N-3-oxotetradec-7z-enoyl-l-homoserine lactone | 0.011971983 | [M+H]+ |
| Isopimpinellin | 0.012117332 | [M+Na]+ |
| Flusilazole | 0.012201302 | [M+H]+ |
| Lnt | 0.012277641 | [M+Na]+ |
| Penitrem a | 0.012475841 | [M+H-C3H8O2]+ |
| Creatine | 0.012479449 | [M+H]+ |
| Benzenesulfonamide, n-(1,1-dimethylethyl)-3-[[5-methyl-2-[[4-(4-methyl-1-piperazinyl)phenyl]amino]-4-pyrimidinyl]amino]- | 0.013363565 | [M+H]+ |
| Cadusafos | 0.013435618 | [2M+H]+ |
| Leu-Tyr-Arg | 0.013611659 | [M+H]+ |
| (-)-sulforaphene | 0.013836725 | [M+H]+ |
| Choline | 0.013973868 | [M]+ |
| Glycochenodeoxycholate | 0.014168333 | [M+Na]+ |
| 11.beta.-hydroxyetiocholanolone | 0.014395535 | [M+H-2H2O]+ |
| N-acetylsulfamethoxazole | 0.014468853 | [M+H]+ |
| Leu-Ile-Arg | 0.014472213 | [M+H]+ |
| 3,4-dimethylmethcathinone | 0.014968102 | [M+H-H2O]+ |
| Epoxomicin | 0.015404649 | [M+H]+ |
| 1-methyladenosine | 0.016422297 | [M+H]+ |
| Pygenic acid c | 0.016473661 | [M+H-H2O]+ |
| All-trans-4-hydroxyretinoic acid | 0.016638902 | [M+H-CH4O3]+ |
| Feruloyl tyramine | 0.017315825 | [M+Na]+ |
| Tyr-Ser-Lys | 0.017430061 | [M+H]+ |
| Nevirapine | 0.017757672 | [M+H]+ |
| Dinaciclib | 0.018160649 | [M+H]+ |
| 3h-imidazo(4,5-f)quinoline, 2-amino-3-methyl- | 0.01819004 | [M+H]+ |
| Nicardipine | 0.018266458 | [M+H]+ |
| Terbuthylazine-tp csaa036479 (lm2) | 0.018446345 | [M+H]+ |
| 4-nitrosodiphenylamine | 0.018700465 | [M+H]+ |
| Cyclocytidine | 0.01871138 | [M+H-H2O]+ |
| Erythromycin (e-mycin) | 0.018889342 | [M+Na]+ |
| Stachydrine | 0.019246391 | [M+H]+ |
| Glandicoline b | 0.019258797 | [M+H]+ |
| Dodecanoic acid, 12-[[(cyclohexylamino)carbonyl]amino]- | 0.01933791 | [M+H-C7H13NO2]+ |
| (+)-chlorpheniramine | 0.019427276 | [M+H]+ |
| Thioetheramide-PC | 0.019654356 | (M+CH3CN+Na)+ |
| Diethyltoluamide | 0.019943986 | [M+H]+ |
| Isopentenyladenosine | 0.019946187 | [M+H]+ |
| Fumonisin b2 | 0.020031348 | [M+Na]+ |
| 5.alpha.-cholest-7-en-3.beta.-ol | 0.020035845 | [M+H-H2O]+ |
| Val-Gly | 0.020423854 | [M+H]+ |
| Lnfp iii | 0.021037771 | [M+K]+ |
| Carnosol | 0.02105577 | [M+H]+ |
| Cytarabine | 0.021114016 | [M+H]+ |
| Valganciclovir | 0.021186395 | [2M+H]+ |
| Anabasine | 0.021437813 | [M+H-NH3]+ |
| Temephos | 0.021502209 | [M+Na]+ |
| L-cystine | 0.021619577 | [M+H]+ |
| Fluazifop-butyl | 0.022183758 | [M+H-C5H10O2]+ |
| Deoxycorticosterone acetate | 0.022245372 | [M+H-H2O]+ |
| Palmitoyl ethanolamide | 0.022364919 | [M+H]+ |
| Val-Ser-Arg | 0.022683996 | [M+2H]2+ |
| 3-methylcytidine | 0.022787941 | [M+H]+ |
| Baccatin iii | 0.023093415 | [M+Na]+ |
| Tetrazolo[1,5-b]pyridazine, 6-(phenylsulfinyl)- | 0.023572037 | [M+H-C4H3N5]+ |
| Lys-Cys | 0.023577761 | [M+H]+ |
| 6h-thieno[3,2-f][1,2,4]triazolo[4,3-a][1,4]diazepine-6-acetic acid, 4-(4-chlorophenyl)-2,3,9-trimethyl-, 1,1-dimethylethyl ester, (6r)- | 0.024215653 | [M+Na]+ |
| Hydrocodone | 0.024228736 | [M+H]+ |
| Glycerophosphocholine | 0.02463266 | [M+H]+ |
| Destruxin a | 0.024698729 | [M+H]+ |
| (-)-riboflavin | 0.024823093 | [M+H]+ |
| Praziquantel | 0.025452659 | [M+H]+ |
| (-)-caryophyllene oxide | 0.025506818 | [2M+H]+ |
| Isodeoxycholic acid | 0.026204503 | [M+Na]+ |
| Propofol .beta.-d-glucuronide | 0.026404826 | [M+H-2H2O]+ |
| Glu-Asn-Arg | 0.026511881 | [M+H]+ |
| N-formylamphetamine | 0.026662536 | [M+H]+ |
| Cytidine | 0.02692305 | [2M+H]+ |
| Miltefosine | 0.02739067 | [M+H-C16H32]+ |
| 2-propenethioamide, 3-[3,5-bis(1,1-dimethylethyl)-4-hydroxyphenyl]-2-cyano-, (2e)- | 0.027758256 | [M+H]+ |
| 19-hydroxytestosterone | 0.028169095 | [M+H-CH4O2]+ |
| 7.alpha.,24(s)-dihydroxycholesterol | 0.028400062 | [M+H-2H2O]+ |
| Estrone sulfate | 0.028978354 | [M+H-H2O]+ |
| Daurisoline | 0.028998373 | [M+H]+ |
| Dichlorvos | 0.02915331 | [2M+H+2i]+ |
| Coproporphyrin I | 0.029184219 | [M+H]+ |
| Pyraclonil | 0.029593874 | [M+H]+ |
| S-adenosyl-l-homocysteine | 0.030788809 | [M+H]+ |
| L-citrulline | 0.030877086 | [M+H]+ |
| Pro-Val-Arg | 0.030965041 | [M+H]+ |
| Granisetron | 0.031235525 | [M+H]+ |
| L-homoarginine | 0.031236499 | [M+H]+ |
| Progesterone 3-peg11-biotin | 0.031341972 | [M+2H]2+ |
| 3-oxazolidinecarboxylic acid, 2,2-dimethyl-4-(1-oxo-2-hexadecyn-1-yl)-, 1,1-dimethylethyl ester, (4s)- | 0.031677327 | [M+Na]+ |
| 1,2-bis(o-octanoyl)-sn-glycerylphophorylcholine | 0.031682125 | [M+Na]+ |
| Fenfluramine | 0.031967648 | [M+H]+ |
| Metaproterenol | 0.033455878 | [M+H]+ |
| Cyclopamine | 0.033592045 | [M+H]+ |
| Enniatin a1 | 0.033623326 | [M+Na]+ |
| Leukotriene c4 | 0.033700267 | [M+H-H2O]+ |
| N-(phenylacetyl)-l-phenylalanine | 0.035769533 | [M+H-CH2O2]+ |
| 13[(2r,3s)-3-pentyloxiranyl]-8z-tridecenoic acid | 0.035992594 | [M+H]+ |
| Oxadiazon | 0.036213888 | [M+H-C3H6+2i]+ |
| 4-aminophenyl-1-thio-.beta.-d-galactopyranoside | 0.036381464 | [M+H-C2H8O4]+ |
| Arginine | 0.03762404 | [M+H]+ |
| 5-methylcytidine | 0.037667961 | [2M+H]+ |
| 1,24-dihydroxyvitamin d3 | 0.037755952 | [M+H-2H2O]+ |
| Betaine | 0.038962308 | [M+H]+ |
| 8-prenylnaringenin | 0.039289506 | [M+H]+ |
| .gamma.-glu-cys | 0.039533695 | [M+H]+ |
| 5-aminovaleric acid | 0.039959786 | [M+H]+ |
| Levorphanol | 0.040565388 | [M+H]+ |
| Chorismic acid | 0.04075166 | [M+H-2H2O]+ |
| Benzamide, n-[5-[2-(3,5-dimethoxyphenyl)ethyl]-1h-pyrazol-3-yl]-4-[(3r,5s)-3,5-dimethyl-1-piperazinyl]-, rel- | 0.040828706 | [M+H]+ |
| 4-chlorobutyric acid ethyl ester | 0.041582161 | [M+H]+ |
| Nis(monooleoylglycero)phosphate (s,r isomer) | 0.041668424 | [M+Na]+ |
| O-succinyl-l-homoserine | 0.042073024 | [M+H]+ |
| 7-benzyl-11,14,20-trimethyl-16-(2-methylpropyl)-10,13-di(propan-2-yl)-17-oxa-1,5,8,11,14-pentazabicyclo[17.3.0]docosane-2,6,9,12,15,18-hexone | 0.042286692 | [M+K]+ |
| Glu-Ser | 0.042346548 | [M+H]+ |
| L-abrine | 0.042538828 | [M+H-CH3NH2]+ |
| 4-chloro-o-toluidine | 0.042631674 | [M+H]+ |
| Bupirimate | 0.043634325 | [M+H]+ |
| Castanospermine | 0.044090056 | [M+H]+ |
| 19(r)-hydroxyprostaglandin f2.alpha. | 0.044287254 | [M+H-H2O]+ |
| Zafirlukast | 0.044348304 | [M+H]+ |
| Trachelanthine | 0.044562682 | [M+H]+ |
| Pyrimidifen | 0.045406646 | [M+H]+ |
| Succinic acid n,n-dimethylhydrazide | 0.045454472 | [M+H-H2O]+ |
| Monoelaidin | 0.045678056 | [M+H-H2O]+ |
| Methyl dihydrojasmonate | 0.04569961 | [M+H-H2O]+ |
| Glutamate conjugated cholic acid | 0.045703992 | [M+Na]+ |
| N-(2-aminoethyl)-5-isoquinolinesulfonamide | 0.046717154 | [M+H]+ |
| Phe-Leu-Lys | 0.047567827 | [M+H]+ |
| Niacinamide | 0.048033628 | [M+H]+ |
| Visnagin | 0.048382186 | [M+H-CH3]+ |
| Pyridaphenthion | 0.049532413 | [M+H-C4H9O2PS]+ |
| Lpc 18:2 | 0.049987955 | [M+H]+ |
| (+)-abscisic acid | 0.000899587 | [M-H-H2O]- |
| 2-phosphonoethylphosphonic acid | 0.001106865 | [M-H]- |
| 2-oleoyl-1-palmitoyl-sn-glycero-3-phosphoserine | 0.001491363 | [M-H]- |
| Ketoprofen .beta.-d-glucuronide | 0.001751799 | [M-H]- |
| 2,3-dihydroxybiphenyl | 0.002090082 | [M-H]- |
| Bisphenol e | 0.002157745 | [M-H]- |
| Desmethylverapamil | 0.00235639 | [M-H]- |
| Anacardic acid | 0.00281546 | [M-H]- |
| Arenobufagin | 0.002825715 | [M-H]- |
| 6-[(3e,6e)-2,5-dihydroxy-4,6-dimethyl-7-(1,2,4-trimethyl-3,6-dioxabicyclo[3.1.0]hexan-4-yl)hepta-3,6-dien-2-yl]-4-methoxy-3,5-dimethylpyran-2-one | 0.002854694 | [M+FA-H]- |
| Cinobufagin | 0.002879246 | [M+FA-H]- |
| Androsterone glucuronide | 0.002960968 | [M-H]- |
| 3,4-dihydroxymandelic acid | 0.003175931 | [2M-H]- |
| 16-phenoxytetranorprostaglandin a2 | 0.003219946 | [M-H]- |
| 8,11-tridecadienoic acid, 13-(3-pentyl-2-oxiranyl)-, (8z,11z)- | 0.003300142 | [M-H-C6H12O]- |
| N-acetyl-s-geranylgeranyl-l-cysteine | 0.003586497 | [M-H]- |
| 3-(n-morpholino)-2-hydroxypropanesulfonic acid | 0.003844957 | [M-H]- |
| Isogentisin | 0.00410748 | [M-H-CH3]- |
| His-Leu | 0.004315418 | [M-H]- |
| Flemiphilippinin a | 0.004766461 | [M-H]- |
| Thiopental | 0.004870703 | [M-H]- |
| 3,5-dimethoxy-4-hydroxycinnamic acid | 0.005098977 | [M-H-CH3]- |
| Folate | 0.005136648 | [M-H-CO2]- |
| Loureirin a | 0.005324504 | [M-H]- |
| Picolinic acid | 0.005509808 | [M-H]- |
| Methanone, [6-hydroxy-1-[2-(4-morpholinyl)ethyl]-1h-indol-3-yl]-1-naphthalenyl- | 0.005743247 | [M-H]- |
| 4-mercaptobenzoic acid | 0.00575853 | [M-H]- |
| Pg 38:6 | 0.005985858 | [M-H]- |
| N-methylleucine | 0.006099032 | [M-H]- |
| 1,3,6-trihydroxy-2-(3-methylbut-2-enyl)xanthen-9-one | 0.006168772 | [M-H]- |
| 4,6-dinitro-o-cresol | 0.006507055 | [M-H-HO]- |
| Diclofop | 0.006660545 | [M-H]- |
| Pregnenolone sulfate | 0.006811297 | [M-H]- |
| Natamycin | 0.006976012 | [M-H-C7H15NO7]- |
| Pentachlorophenol | 0.007870243 | [M-H]- |
| L-homocysteic acid | 0.008030336 | [M-H]- |
| Benzeneacetic acid, 5-[2-[4-(ethoxycarbonyl)phenyl]diazenyl]-2-hydroxy- | 0.008039913 | [M-H]- |
| sn-Glycerol 3-phosphoethanolamine | 0.008119892 | [M-H]- |
| O-phosphoethanolamine | 0.008715138 | [M-H]- |
| Pantothenate | 0.009089975 | [M-H]- |
| 5-hydroxytryptophol | 0.009539201 | [M-H]- |
| Xanthylic acid (xmp) | 0.009648494 | [M-H]- |
| Acamprosate | 0.00983522 | [M-H]- |
| Thiorphan | 0.010153457 | [M-H]- |
| 1.alpha.-methyl-5.alpha.-androstan-3.alpha.,17.beta.-diol glucuronide | 0.010204726 | [M-H]- |
| Beta-alanine | 0.010639704 | [M-H]- |
| 4-hydroxyisoleucine | 0.010702179 | [M-H]- |
| Dl-4-hydroxy-3-methoxymandelic acid | 0.011474524 | [2M-H]- |
| Trans-dehydroandrosterone | 0.011559684 | [M-H]- |
| Pseudouridine | 0.012846507 | [M-H]- |
| Phosphoenolpyruvate | 0.013017273 | [2M-H]- |
| 7s,8,17s-trihydroxydocosa-4z,9e,11e,13z,15e,19z-hexaenoic acid | 0.01302503 | [M-H]- |
| Andrastin d | 0.01397808 | [M-H]- |
| O,o-diethyl phosphate | 0.014403265 | [M-H]- |
| 2'-chloro-2-hydroxy-5-methylbenzophenone | 0.01556385 | [M-H]- |
| Harmane | 0.015710257 | [M-H]- |
| Adenosine 5'-phosphosulfate | 0.015711681 | [M-H-O3S]- |
| Candesartan | 0.015725862 | [M-H]- |
| Undecanamide, n-cyclopropyl-11-(2-hexyl-5-hydroxyphenoxy)- | 0.015932936 | [M-H]- |
| Gardnerine | 0.015999207 | [M-H]- |
| Periplogenin | 0.016270905 | [M-H]- |
| Methyl 3-[3,4-dihydroxy-5-(3-methylbut-2-enyl)phenyl]-2-[[4-hydroxy-3-(3-methylbut-2-enyl)phenyl]methyl]-4-methoxy-5-oxofuran-2-carboxylate | 0.016337535 | [M-H]- |
| Formylanthranilic acid | 0.016351244 | [M-H]- |
| 2-ketobutyric acid | 0.01663385 | [M-H]- |
| (z)-9,12,13-trihydroxyoctadec-15-enoic acid | 0.01735634 | [M-H]- |
| L-homocystine | 0.018377798 | [M-H-C4H9SO2N]- |
| Neobavaisoflavone | 0.018711669 | [M-H]- |
| Pantothenic acid | 0.018811696 | [2M-H]- |
| Nepodin | 0.019340671 | [M-H]- |
| 2,4-dimethylthiazole-5-carboxylic acid | 0.019990882 | [M-H]- |
| Iminodiacetic acid | 0.019996449 | [M-H-H2O]- |
| 4-imidazolidineheptanoic acid, 3-[(2-cyclohexyl-2-hydroxyethyl)amino]-2,5-dioxo-1-(phenylmethyl)- | 0.020404031 | [M-H-C8H17NO]- |
| N-(2-phosphate-1r-methylethyl)-5z,8z,11z,14z-eicosatetraenamide | 0.02070175 | [M-H]- |
| Neochlorogenic acid | 0.020918193 | [M-H-C9H6O3]- |
| Chenodeoxycholic acid 24-acyl-.beta.-d-glucuronide | 0.021157338 | [M-H]- |
| Lpe 18:2 | 0.021723322 | [M-H]- |
| Rhein | 0.022183321 | [M-H]- |
| Phosphocreatine | 0.022488866 | [M-H]- |
| Methanone, [1-(5-fluoropentyl)-2-hydroxy-1h-indol-3-yl]-1-naphthalenyl- | 0.022564935 | [M-H]- |
| Oleanolic acid | 0.022806169 | [M-H]- |
| 6a,12a-didehydroamorphigenin | 0.022887118 | [M-H]- |
| Senegenin | 0.023258695 | [M-H]- |
| Dehydro-l-(+)-ascorbic acid dimer | 0.023886508 | [M-H-C7H8O7]- |
| P-coumaric acid | 0.025089127 | [M-H-H2O]- |
| Mitoxantrone | 0.02525293 | [M-H]- |
| Biocytin | 0.025484918 | [M-H]- |
| Pro-tyr | 0.025593106 | [M-H]- |
| Xanthine | 0.027259977 | [M-H]- |
| Isoimperatorin | 0.027529103 | [M-H]- |
| Pg 34:1 | 0.027620622 | [M-H]- |
| 2-hydroxyphenylacetic acid | 0.028367196 | [M-H]- |
| L-homocitrulline | 0.028638254 | [M-H]- |
| (-)-epigallocatechin | 0.029094144 | [M-H]- |
| 2-chlorobenzoic acid | 0.029249211 | [M-H]- |
| Trans-2-hydroxycinnamic acid | 0.029615999 | [M-H]- |
| Phytanic acid | 0.030290375 | [M-H]- |
| Benzoic acid, 3-[(e)-[(3ar,4s,5s,6ar)-4-[(1e,3r)-3-cyclohexyl-3-hydroxy-1-propen-1-yl]hexahydro-5-hydroxy-2(1h)-pentalenylidene]methyl]-, rel- | 0.030342783 | [M-H]- |
| Glycine | 0.030368999 | [M-H]- |
| Benzylphosphonic acid | 0.030640479 | [M-H-H2O]- |
| Dodecanedioic acid | 0.032523434 | [M-H]- |
| N-oleoylglycine | 0.032728149 | [M-H]- |
| Benzoic acid, 3-[[(3-carboxycyclohexyl)amino]carbonyl]-4-[3-[4-[4-(cyclohexyloxy)butoxy]phenyl]propoxy]- | 0.033155141 | [M+Cl]- |
| Xanthosine | 0.033255383 | [M-H]- |
| 3.beta.,7.alpha.-dihydroxy-5-cholestenoic acid | 0.03358223 | [M-H]- |
| 6-phosphogluconic acid | 0.033591431 | [M-H]- |
| Linoleoylglycine | 0.034555119 | [M-H]- |
| Glyceric acid | 0.034604442 | [M-H]- |
| Pg 40:8 | 0.034615344 | [M-H]- |
| L-Histidinol phosphate | 0.035025305 | [M-H]- |
| Telmisartan | 0.035122206 | [M-H-CO2]- |
| Oligomycin a | 0.035293643 | [M-H-C11H20O4]- |
| Indole-3-butyric acid | 0.03535196 | [M-H]- |
| 3-dehydrocholic acid | 0.036659726 | [M-H]- |
| 7(s),17(s)-dihydroxy-8(e),10(z),13(z),15(e),19(z)-docosapentaenoic acid | 0.037641464 | [M-H-H2O]- |
| Forsythoside e | 0.038720714 | [M-H]- |
| Cysteine | 0.038911063 | [M-H]- |
| Mitragynine | 0.039271646 | [M-H]- |
| N-arachidonoyl-l-alanine | 0.039598913 | [M-H]- |
| Arachidonic acid (peroxide free) | 0.040852556 | [M-H]- |
| (1-acetyloxy-3-hydroxy-6,8a-dimethyl-7-oxo-3-propan-2-yl-2,3a,4,8-tetrahydro-1h-azulen-4-yl) 4-hydroxybenzoate | 0.041217257 | [M-H]- |
| L-gulono-1,4-lactone | 0.041235473 | [M+OH]- |
| Resorcinolnaphthalein | 0.041683782 | [M-H]- |
| L-cysteine-glutathione disulfide | 0.04290228 | [M-H-H2O]- |
| .beta.-glycerophosphate | 0.043490581 | [M-H]- |
| 3-(cyclohexylamino)-2-hydroxy-1-propanesulfonic acid | 0.043499889 | [M-H]- |
| Osmanthuside h | 0.043717152 | [M-H]- |
| 4-methylumbelliferyl .beta.-d-glucuronide | 0.044216016 | [M-H]- |
| Linoleic acid | 0.044493666 | [M-H]- |
| Lithosprmoside | 0.046537259 | [M-H]- |
| (2r,3s,4s,5r,6r)-2-[[(2s,3r,4r)-3,4-dihydroxy-4-(hydroxymethyl)oxolan-2-yl]oxymethyl]-6-[4-(4-hydroxyphenyl)butan-2-yloxy]oxane-3,4,5-triol | 0.046707654 | [M-H]- |
| Adenosine 5'-diphosphate | 0.047838985 | [M-H]- |
| 21-Hydroxypregnenolone | 0.049092368 | [M-H]- |
| 5-hydroxy-3,4-dihydro-2(1h)-quinolinone | 0.049568541 | [M-H]- |
| 1-(1,2-dihexadecanoylphosphatidyl)inositol-3-phosphate | 0.04987262 | [M-2H]2- |
