# Supplementary material for: Different lysine-to-methionine ratios in a low-protein diet affect the microbiome and metabolome, influencing the jejunal barrier function in Tibetan sheep
Source: Front Microbiol. 2025 Feb 12;16:1441143. doi: 10.3389/fmicb.2025.1441143 (PMC11861081; doi:10.3389/fmicb.2025.1441143)
Supplement: Supplementary file 2 [file Image_1.pdf]

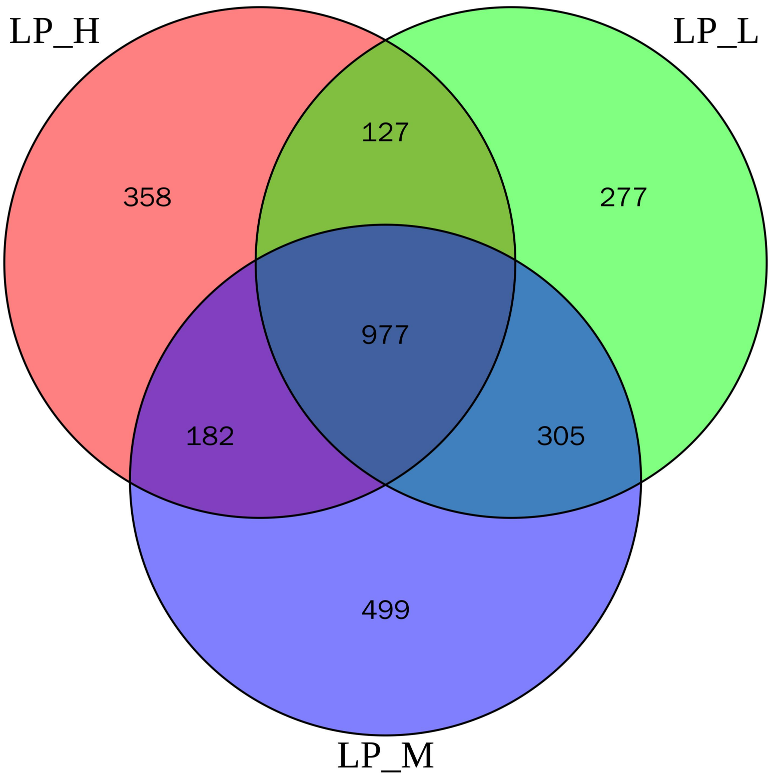

Figure S1. Venn diagram of operational taxonomic units (OTUs) of each group.

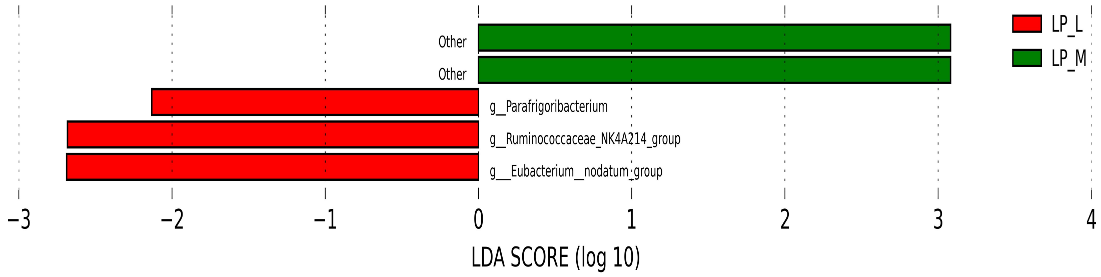

Figure S2. Linear discriminant analysis effect size (LEfSe) analysis of each group.

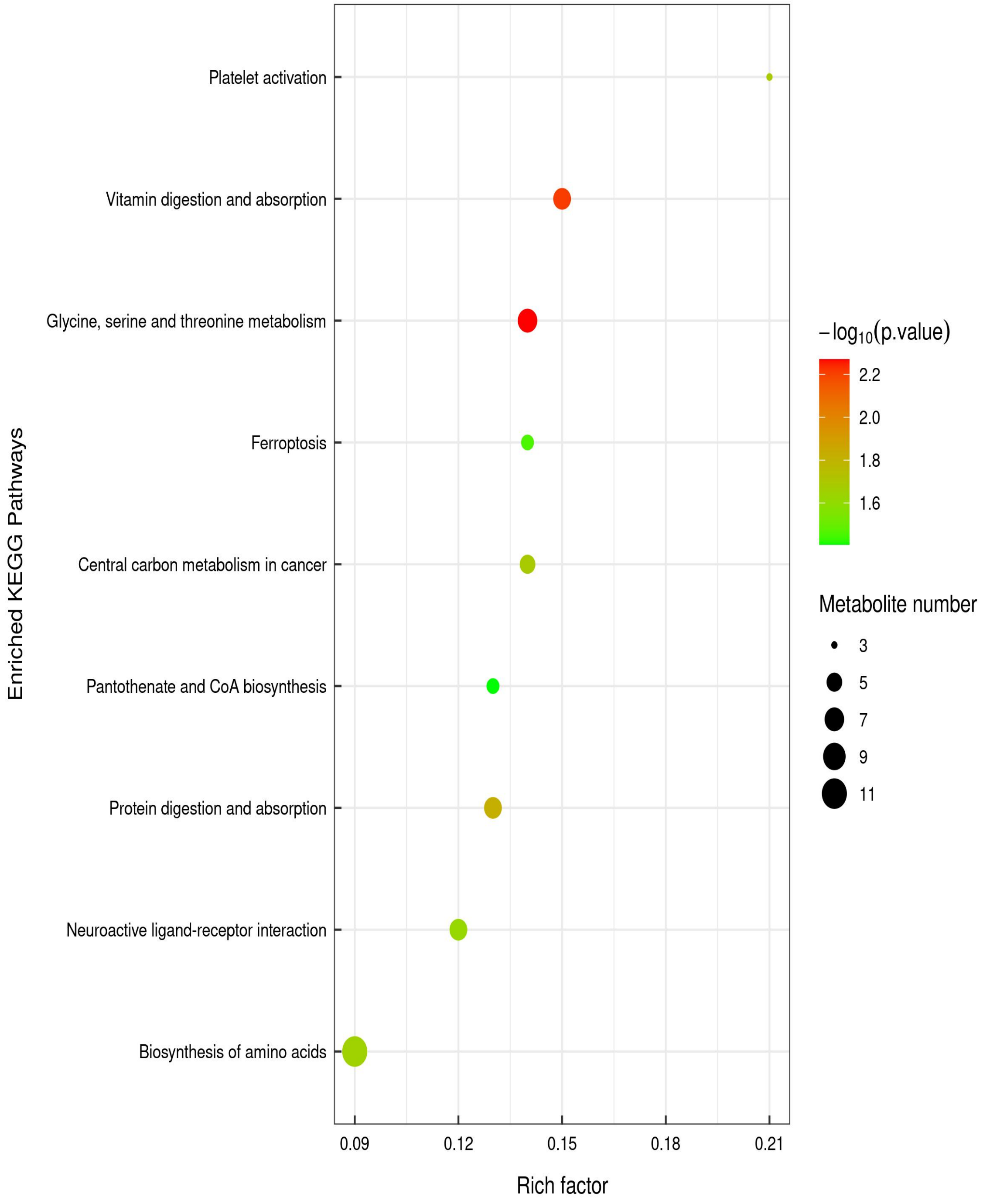

Figure S3. The bubble chart of enrichment analysis for metabolic pathways.

The manipulated metabolic pathways are based on the analysis of different metabolites among three treatments following the KEGG pathway database. The metabolome view shows all matched pathways according to the P-values from the pathway enrichment analysis and impact values from the topology analysis. The node colors varied from green to red, indicating that the metabolites have different levels of significance.
